# Metabolic Fluxes of Nitrogen and Pyrophosphate in Chemostat Cultures of Clostridium thermocellum and Thermoanaerobacterium saccharolyticum

**DOI:** 10.1128/AEM.01795-20

**Published:** 2020-11-10

**Authors:** Evert K. Holwerda, Jilai Zhou, Shuen Hon, David M. Stevenson, Daniel Amador-Noguez, Lee R. Lynd, Johannes P. van Dijken

**Affiliations:** a Thayer School of Engineering, Dartmouth College, Hanover, New Hampshire, USA; b The Center for Bioenergy Innovation, Oak Ridge National Laboratory, Oak Ridge, Tennessee, USA; c Department of Bacteriology, University of Wisconsin-Madison, Madison, Wisconsin, USA; d Delft University of Technology, Delft, The Netherlands; University of Michigan-Ann Arbor

**Keywords:** pyrophosphate, glycolysis, amino acid excretion, *Clostridium thermocellum*, *Thermoanaerobacterium saccharolyticum*, chemostat culture, carbon limitation, nitrogen limitation

## Abstract

Clostridium thermocellum and Thermoanaerobacterium saccharolyticum were grown in cellobiose-limited chemostat cultures at a fixed dilution rate. C. thermocellum produced acetate, ethanol, formate, and lactate. Surprisingly, and in contrast to batch cultures, in cellobiose-limited chemostat cultures of T. saccharolyticum, ethanol was the main fermentation product. Enzyme assays confirmed that in C. thermocellum, glycolysis proceeds via pyrophosphate (PP_i_)-dependent phosphofructokinase (PFK), pyruvate-phosphate dikinase (PPDK), as well as a malate shunt for the conversion of phosphoenolpyruvate (PEP) to pyruvate. Pyruvate kinase activity was not detectable. In T. saccharolyticum, ATP but not PP_i_ served as cofactor for the PFK reaction. High activities of both pyruvate kinase and PPDK were present, whereas the activities of a malate shunt enzymes were low in T. saccharolyticum. In C. thermocellum, glycolysis via PP_i_-PFK and PPDK obeys the equation glucose + 5 NDP + 3 PP_i_ → 2 pyruvate + 5 NTP + P_i_ (where NDP is nucleoside diphosphate and NTP is nucleoside triphosphate). Metabolic flux analysis of chemostat data with the wild type and a deletion mutant of the proton-pumping pyrophosphatase showed that a PP_i_-generating mechanism must be present that operates according to ATP + P_i_ → ADP + PP_i_. Both organisms also produced significant amounts of amino acids in cellobiose-limited cultures. It was anticipated that this phenomenon would be suppressed by growth under nitrogen limitation. Surprisingly, nitrogen-limited chemostat cultivation of wild-type C. thermocellum revealed a bottleneck in pyruvate oxidation, as large amounts of pyruvate and amino acids, mainly valine, were excreted; up to 50% of the nitrogen consumed was excreted again as amino acids.

**IMPORTANCE** This study discusses the fate of pyrophosphate in the metabolism of two thermophilic anaerobes that lack a soluble irreversible pyrophosphatase as present in Escherichia coli but instead use a reversible membrane-bound proton-pumping enzyme. In such organisms, the charging of tRNA with amino acids may become more reversible. This may contribute to the observed excretion of amino acids during sugar fermentation by Clostridium thermocellum and Thermoanaerobacterium saccharolyticum. Calculation of the energetic advantage of reversible pyrophosphate-dependent glycolysis, as occurs in Clostridium thermocellum, could not be properly evaluated, as currently available genome-scale models neglect the anabolic generation of pyrophosphate in, for example, polymerization of amino acids to protein. This anabolic pyrophosphate replaces ATP and thus saves energy. Its amount is, however, too small to cover the pyrophosphate requirement of sugar catabolism in glycolysis. Consequently, pyrophosphate for catabolism is generated according to ATP + P_i_ → ADP + PP_i_.

## Abstract (precis)

This study discusses the fate of pyrophosphate in the metabolism of two thermophilic anaerobes that lack a soluble irreversible pyrophosphatase as present in Escherichia coli but instead use a reversible membrane-bound proton-pumping enzyme. In such organisms, the charging of tRNA with amino acids may become more reversible. This may contribute to the observed excretion of amino acids during sugar fermentation by Clostridium thermocellum and Thermoanaerobacterium saccharolyticum. Calculation of the energetic advantage of reversible pyrophosphate-dependent glycolysis, as occurs in Clostridium thermocellum, could not be properly evaluated, as currently available genome-scale models neglect the anabolic generation of pyrophosphate in, for example, polymerization of amino acids to protein. This anabolic pyrophosphate replaces ATP and thus saves energy. Its amount is, however, too small to cover the pyrophosphate requirement of sugar catabolism in glycolysis. Consequently, pyrophosphate for catabolism is generated according to ATP + P_i_ → ADP + PP_i_.

## INTRODUCTION

In the past 2 decades, the thermophilic anaerobic bacteria Clostridium thermocellum and Thermoanaerobacterium saccharolyticum have received much attention in view of their possible application in the conversion of lignocellulosic biomass to ethanol. Fermentation products of both organisms are acetate, ethanol, formate, lactate, and H_2_. T. saccharolyticum has been engineered into an ethanologenic organism producing ethanol at high yields at titers in excess of 60 g/liter (1). Engineering of C. thermocellum into an ethanologenic organism has been more difficult than in T. saccharolyticum. To date, ethanol production from cellulose with engineered strains has not exceeded titers of 25 to 30 g/liter (2, 3). Current research focuses on the replacement of the enzymes of glycolysis (the conversion of glucose to pyruvate) and redox enzymes of C. thermocellum with those of T. saccharolyticum to achieve higher ethanol titers (4–6), as well as other approaches.

Glycolysis in C. thermocellum resembles that of Entamoeba histolytica and other parasitic protists (7, 8) in being reversible due to pyrophosphate-dependent phosphofructokinase (PP_i_-PFK) and pyruvate-phosphate dikinase (PPDK), a situation also encountered in plants and algae (9–11). The involvement of these enzymes results in the overall stoichiometry:

$$\text{glucose }+\text{ 5 ADP }+3{\text{ PP}}_{\text{i}}\to \text{ 2 pyruvate }+\text{ 5 ATP }+{\text{ P}}_{\text{i}}.$$

A major contribution to this apparent high ATP yield of the PP_i_-dependent glycolysis arises from the PPDK reaction:

$$\text{PEP }+\text{ AMP }+{\text{PP}}_{\text{i}}\to \text{pyruvate }+\text{ ATP }+{\text{ P}}_{\text{i}}\;\left(\text{PPDK}\right)$$

$$\frac{2\;\text{ADP}\to \text{AMP}+\text{ATP}(\text{adenylate kinase})}{{\text{PEP}+2\;\text{ADP}+\text{PP}}_{\text{i}}\to {\text{pyruvate}+2\;\text{ATP}+\text{P}}_{\text{i}}}+$$

where PEP is phosphoenolpyruvate.

In several publications in which pyrophosphate-dependent glycolysis with PP_i_-PFK and PPDK is discussed, it is neglected that the large catabolic glycolytic flux cannot be sustained by the amount of PP_i_ generated as a product in biosynthetic reactions (12). Furthermore, glycolysis with an output of 5 ATP/glucose would have no thermodynamic driving force. An ATP yield higher than the usual 2 ATP in the conversion of glucose to pyruvate is only possible if a membrane-bound pyrophosphatase would act as a PP_i_ synthase via the proton motive force (13, 14). For example, if the PP_i_-to-ATP ratio would equal 2, then 3.5 ATP/glucose would be possible (15).

In this study, the role of PP_i_-PFK and PPDK in C. thermocellum and T. saccharolyticum is addressed in relation to the PP_i_ requirement and ATP yield of glycolysis during growth of both organisms under the same conditions in cellobiose-limited chemostat cultures in a defined medium. Particular attention is paid to the excretion of pyruvate-derived amino acids as overflow products of glycolysis in C. thermocellum by nitrogen-limited chemostat cultures.

## RESULTS

### Chemostat cultivation.

**FIG 1:**
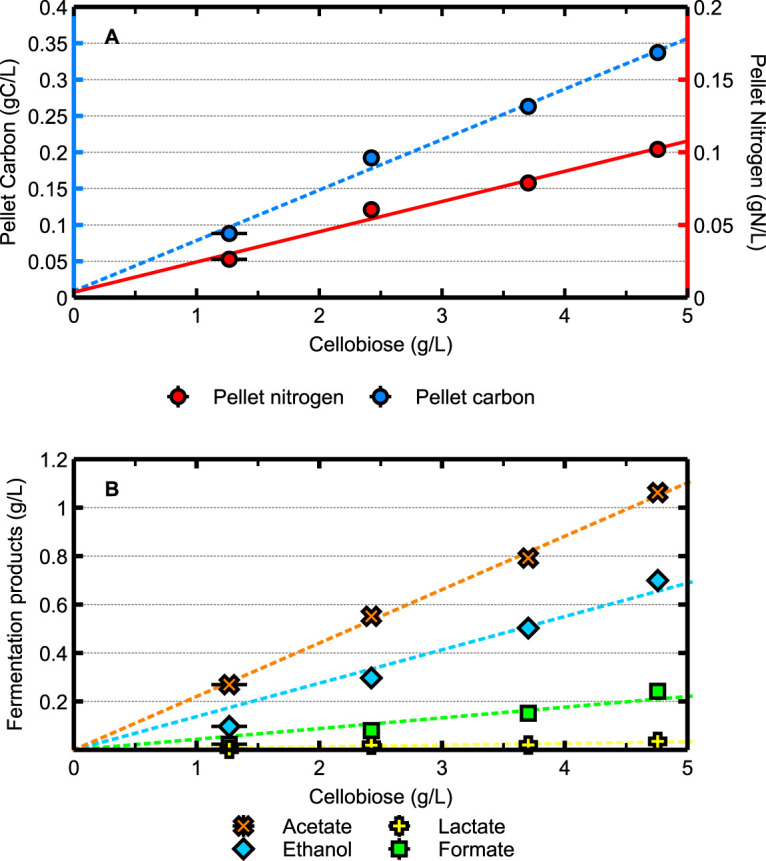
Concentrations of biomass (A) and fermentation products (B) in chemostat cultures of Clostridium thermocellum growing at various cellobiose concentrations at *D* = 0.1 h^−1^, where *D* represents the dilution rate. The slopes of the fit lines in panel A for pellet carbon and nitrogen are 6.95 × 10^−2^ and 2.08 × 10^−3,^ respectively, resulting in a C/N ratio of 3.34 g C/g N. The error bars represent one standard deviation.

Steady-state chemostat cultures were used to study glycolytic fluxes because, in such cultures, all metabolic fluxes are constant. Chemostats also allow a comparison of the physiology of organisms under the same growth conditions. Rigorous testing of the nature of the growth limitation in the chemostat cultures was carried out. The defined medium described in Materials and Methods sustained carbon- and energy-limited growth of both C. thermocellum (Fig. 1; Table 1) and T. saccharolyticum (data not shown) up to 5 g cellobiose/liter. The concentrations of biomass and fermentation products in steady-state cultures were linearly proportional to the inflowing cellobiose concentration, and residual cellobiose was below the detection limit. Notable differences between the two organisms are the much lower biomass concentration of T. saccharolyticum and the very high ethanol concentration amounting to 94% of the total pyruvate flux (Table 1). This contrasts its behavior in batch culture where, apart from ethanol, also acetate, lactate, and formate are major fermentation products. Poor carbon balances were obtained for both organisms (Table 1), which prevents the calculation of the ATP demand for biomass synthesis. Furthermore, apart from the regular fermentation products, both organisms also produced considerable amounts of extracellular protein and amino acids. T. saccharolyticum produced mainly alanine, glutamate, and threonine, whereas, in C. thermocellum, valine replaced threonine as the main amino acid (Table 2).

**TABLE 1:** Carbon balance of C. thermocellum and T. saccharolyticum in cellobiose-limited cultures at *D* = 0.1 h^−1^^*a*^

Component	Concn (mean ± SD [mg/liter])	
	Clostridium thermocellum	Thermoanaerobacterium saccharolyticum
Cell nitrogen	102.0 ± 1.2	39.3 ± 1.1
Cell carbon	337.3 ± 2.2	129.2 ± 2.6
Acetate	1,062.4 ±16.0	47.6 ± 0.7
Ethanol	699.6 ± 22.2	1,876.4 ± 2.3
Lactate	35.3 ± 3.3	129.8 ± 6.1
Formate	241.8 ± 3.6	12.3 ± 0.4
Glucose	20.2 ± 1.0	3.8 ± 0.1
Pyruvate	15.4 ± 2.3	7.6 ± 1.5
Isobutanol	25.3 ± 0.7	ND^*b*^
Excreted amino acids	90.8	110.7
Supernatant protein	103.5 ± 5.7	95.4 ± 17.6
CO_2_	1,254.0	1,815.9
Cellobiose in feed	4,757.2 ± 8.0	4,755.2 ± 8.2
Residual cellobiose	ND	ND
Carbon recovery (%)^*c*^	84.0	88.8

a CO_2_ values are based on the sum of the amounts of acetate, ethanol, and twice the amount of isobutanol minus the amount of formate in moles.

b ND, not detected.

c For the calculation of carbon recovery, 0.532 g C/g protein was used.

**TABLE 2:** Concentrations of individual amino acids in cellobiose-limited chemostat cultures at *D* = 0.1 h^−1^ for C. thermocellum and T. saccharolyticum

Amino acid	Concn (mg/liter)	
	*C. thermocellum*	*T. saccharolyticum*
Alanine	15.0	38.5
Asparagine	0.5	0.2
Aspartic acid	1.7	6.4
Glutamic acid	30.0	28.7
Glutamine	0.3	3.1
Histidine	0.0	0.0
Isoleucine	4.0	1.3
Leucine	3.0	1.9
Lysine	6.5	2.9
Methionine	0.0	0.2
Phenylalanine	1.0	0.0
Proline	4.3	5.1
Serine	4.6	3.1
Threonine	4.0	16.6
Tryptophan	0.3	0.0
Tyrosine	0.6	0.1
Valine	15.0	2.6
Total	90.8	110.7
Total carbon (mg C/liter)	40.5	45.0
Total nitrogen (mg N/liter)	20.6	23.4

### Activities of glycolytic enzymes.

**FIG 2:**
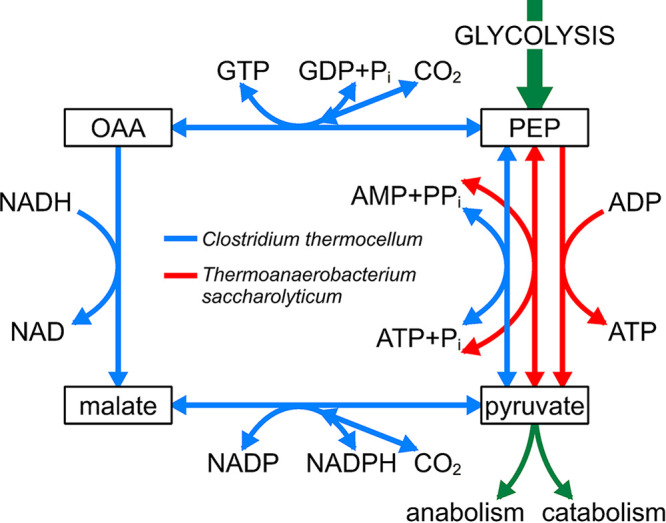
Conversion of PEP to pyruvate in C. thermocellum and T. saccharolyticum. The equilibrium of the malate shunt is toward pyruvate formation, and the equilibrium of the malate dehydrogenase reaction is strongly toward malate formation. With purified enzyme, the reaction appeared irreversible at pH 7.0 (30). The catalytic efficiency (*k*_cat_/*K_m_*) of malate dehydrogenase with NADPH is 12-fold lower than with NADH. Malic enzyme is specific for NADP. The catalytic efficiency of the enzyme in the forward reaction is 16-fold higher than the reverse reaction (30). OAA is oxaloacetic acid.

In C. thermocellum, cellobiose is transported via an ABC transporter (16). Internalized cellobiose is hydrolyzed to glucose and glucose-1-phosphate by cellobiose phosphorylase (EC 2.4.1.20) (17), but cellobiase is present also (18). In T. saccharolyticum, cellobiose transport probably occurs via a phosphotransferase system (PTS) (19), resulting in formation of cellobiose-6-phosphate, which is hydrolyzed to glucose and glucose-6-phosphate by cellobiose-phosphate hydrolyase (EC 3.2.1.86). A comparison of the activities of the glycolytic enzymes is presented in Table 3. Several differences exist between the two organisms. Glucokinase is GTP dependent in C. thermocellum but ATP dependent in T. saccharolyticum. Phosphofructokinase is pyrophosphate dependent in C. thermocellum, whereas the T. saccharolyticum enzyme uses ATP. In addition to PPDK, high activities of the malate shunt enzymes phosphoenolpyruvate (PEP) carboxykinase, malate dehydrogenase, and malic enzyme (ME) were found in C. thermocellum. In T. saccharolyticum, high activities of both pyruvate kinase and PPDK were detected, whereas activities of malate shunt enzymes (Fig. 2) were low (Table 3). Compared to the enzyme activities in anaerobic glucose-limited chemostat cultures of S. cerevisiae grown at the same dilution rate (20), the 15-fold-lower activity of pyruvate kinase in T. saccharolyticum is especially noteworthy. In C. thermocellum, high activities of NADP-linked glutamate dehydrogenase were present. This enzyme was not detected in T. saccharolyticum. The nature of ammonium-assimilating enzymes in this organism was not further investigated. The difference in ammonium assimilation may, however, be relevant for attempts to turn C. thermocellum into a T. saccharolyticum-like organism, which has been engineered to reach high ethanol titers (21, 22).

**TABLE 3:** Enzyme activities in the core metabolism of C. thermocellum and T. saccharolyticum in cellobiose-limited cultures at *D* = 0.1 h^−1^

Enzyme	Cofactor	Enzyme activities (mean ± SD [μmol/mg protein/min])	
		*C. thermocellum*	*T. saccharolyticum*
Glucokinase	GTP	0.71 ± 0.11	0.07 ± 0.02
	ATP	0.06 ± 0.00	0.84 ± 0.05
	PEP	<0.01	<0.01
Phosphofructokinase	PP_i_	1.20 ± 0.28	<0.01
	ATP	<0.01	0.55 ± 0.05
Phosphoglycerate kinase	GDP	2.37 ± 0.14	0.35 ± 0.02
	ADP	3.21 ± 0.28	6.70 ± 1.37
GAP dehydrogenase	NAD	3.86 ± 0.57	11.68 ± 2.68
	NADP	<0.01	0.07 ± 0.02
PEP carboxykinase	ADP	0.16 ± 0.03	0.49 ± 0.02
	GDP	4.80 ± 0.26	0.11 ± 0.04
	P_i_	<0.01	<0.01
Pyruvate kinase	ADP	<0.01	0.65 ± 0.04
Pyruvate phosphate dikinase	AMP+PP_i_	0.19 ± 0.03	0.86 ± 0.09
Malate dehydrogenase	NADH	3.79 ± 0.85	<0.01
	NADPH	0.24 ± 0.05	0.07 ± 0.01
Malic enzyme	NAD	0.05 ± 0.01	0.06 ± 0.01
	NADP	2.78 ± 0.44	0.07 ± 0.01
Glucose-6-phosphate dehydrogenase	NADP	<0.01	0.03 ± 0.01
6-phosphogluconate dehydrogenase	NADP	<0.01	0.05 ± 0.01
Isocitrate dehydrogenase	NAD	<0.01	<0.01
	NADP	1.05 ± 0.07	0.91 ± 0.19
Glutamate dehydrogenase	NAD	<0.01	<0.01
	NADP	2.34 ± 0.35	<0.01

### Generation of NADPH.

In C. thermocellum, the enzymes of the hexose monophosphate (HMP) pathway, glucose-6-P dehydrogenase and 6-phosphogluconate dehydrogenase, were not annotated, and corresponding activities were not detectable. In this organism, ME probably contributes to NADPH synthesis (Table 3). Another source of NADPH is the electron-bifurcating NfnAB complex (5).

$${\text{ferredoxin}}_{\text{red}}+\text{ NADH }+\text{ 2 NADP}\to {\text{ferredoxin}}_{\text{ox}}+\text{ NAD }+\text{ 2 NADPH}$$

Low activities of the HMP pathway enzymes were detected in T. saccharolyticum (Table 3). However, a key enzyme of this pathway, 6-phosphogluconolactonase (EC 3.1.1.31), is not annotated in this organism. Although NfnAB can be a source of NADPH in both C. thermocellum and T. saccharolyticum, it is not the sole mechanism in these organisms, as deletion mutants are still viable (23). Also, isocitrate dehydrogenase cannot be the sole source of NADPH in both organisms, as the generation of NADPH is stoichiometrically coupled to the formation of alpha-ketoglutarate. If isocitrate dehydrogenase was the sole source of NADPH for all transamination reactions with glutamate as an ammonium donor for ketoacids, this would lead to accumulation of alpha-ketoglutarate.

### Role of PPDK in glycolysis.

Both C. thermocellum and T. saccharolyticum possess two options for the conversion for the final reaction in glycolysis: the conversion of PEP to pyruvate (Fig. 2). In C. thermocellum, this reaction can be catalyzed by a malate shunt and PPDK, whereas in T. saccharolyticum, pyruvate kinase and PPDK are present. PPDK can be deleted in batch cultures of both organisms without major changes in biomass formation and yields of the various fermentation products (12, 19, 24). Apparently, PPDK activity is not essential in batch cultures of both organisms. However, this does not mean that the enzyme is not important for the wild type. From a dynamic ^13^C-labeling study, it was concluded that in cellobiose-grown batch cultures of wild-type C. thermocellum, PPDK is responsible for two-thirds of the flux from PEP to pyruvate, whereas one-third of the pyruvate is formed via the malate shunt (24). Similarly, whereas PPDK is nonessential in batch cultures of cellobiose-grown T. saccharolyticum (19), this does not mean that it is not functional in this or other growth conditions. In this respect, it is striking that in cellobiose-limited cultures of T. saccharolyticum, the PPDK activity is even higher than pyruvate kinase (Table 3), contrary to batch cultures (19).

### Generation of pyrophosphate.

In both C. thermocellum and T. saccharolyticum, a soluble pyrophosphatase, as occurs in Escherichia coli, is absent. Instead, a membrane-bound proton-pumping pyrophosphatase is present (Fig. S1 in the supplemental material). This enzyme can also act as a PP_i_ synthase by using the proton-motive force (13–15) and may therefore be a source of pyrophosphate in glycolysis. To elucidate its possible contribution to the ATP yield of the PP_i_-dependent glycolysis in C. thermocellum, it was decided to delete the gene and to investigate the effect of this deletion on the biomass yield. It was anticipated that if the pyrophosphatase was a major source of PP_i_, with a PP_i_-to-ATP ratio higher than unity, its deletion should lead to a decrease in the biomass yield. The membrane-bound pyrophosphatase appeared to be a nonessential enzyme. Furthermore, the deletion strain LL1639 exhibited the same biomass and product yields as wild-type and parental strain in chemostat cultures (Table S2). Either the pyrophosphatase-negative mutant has an alternative mechanism for PP_i_ synthesis, which has the same ATP requirement as the pyrophosphatase-positive wild-type, or pyrophosphatase has a negligible contribution in PP_i_ synthesis.

### Excretion of amino acids by C. thermocellum.

**FIG 3:**
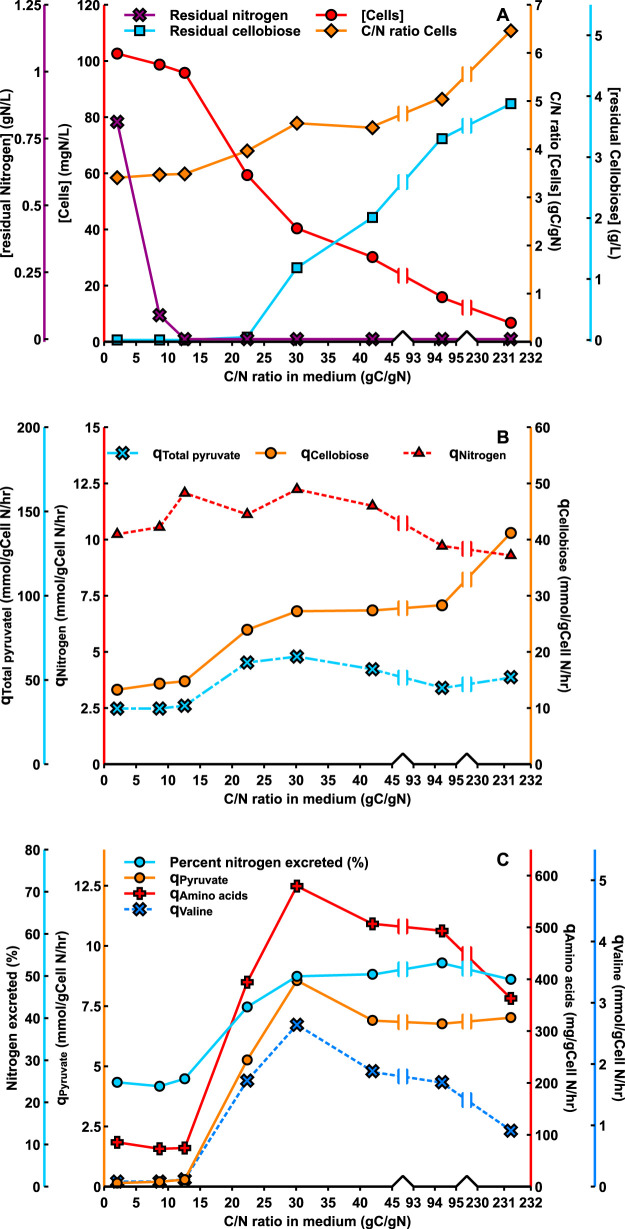
Physiology of C. thermocellum as a function of the C/N ratio in the medium feed during growth on cellobiose with urea as nitrogen source. (A) At and below a C/N ratio of 12.6, cultures were cellobiose limited. Between C/N ratios of 12.6 and 22.5, cultures were limited for both cellobiose and urea, and above this C/N ratio, cultures were exclusively nitrogen limited as evidenced by residual cellobiose in the culture. (B and C) Fluxes are expressed as millimoles per gram cell nitrogen per hour, except for amino acids (aa), which are expressed as milligrams aa per gram cell nitrogen per hour.

C. thermocellum excretes pyruvate and the pyruvate-derived amino acids valine and alanine when growing on excess cellulose (25). This even occurred in carbon (cellobiose)-limited cultures (Tables 1 and 2), a growth condition not associated with overflow metabolism in microorganisms. The cause of this unusual phenomenon is apparently a surplus of pyruvate and NADPH, both of which are required for amino acid synthesis. It was therefore expected that growth under nitrogen limitation would suppress amino acid excretion. To investigate this assumption, C. thermocellum was grown at a wide range of different C/N ratios in the medium with a fixed cellobiose concentration at various concentrations of urea as a nitrogen source. The results of the analysis of such steady-state cultures are presented in Fig. 3, Table 4, and Tables S3, S4, S5, and S6. At C/N ratios of the medium, 2.0 and 8.7 g C/g N, the cultures were cellobiose limited. At a ratio of 12.6, a dual limitation of cellobiose and urea existed, as both cellobiose and urea were below the detection limit (Fig. 3A and Table S3). When the C/N ratio was further increased to 22.4, cellobiose became detectable, and the culture was limited by nitrogen only; no residual urea or ammonia was detectable. A further increase in the C/N ratio resulted in progressively more residual cellobiose and lower biomass concentrations. The C/N ratio of the biomass increased with increasing C/N ratios in the medium, indicative for accumulation of a reserve polymer, probably glycogen (Fig. 3A).

Figures 3B and C present the fluxes of sugar consumption and product formation as millimole per gram biomass nitrogen per hour. This expression was chosen rather than specific rates per gram biomass because the C/N ratio of the biomass changes during nitrogen limitation (Fig. 3A). Therefore, fluxes based on cellular nitrogen better represent the activity of the cellular metabolic machinery (i.e., proteins) than biomass dry weight. The specific rate of cellobiose consumption increased with increasing medium C/N ratio above 12.6 (Fig. 3B; Table 4), whereas the specific nitrogen consumption rates remained approximately constant. The flux of excreted pyruvate and pyruvate-derived products (q_Total pyruvate_) doubled when nitrogen became limiting and decreased at higher C/N ratios in the feed medium (Fig. 3B).

**TABLE 4:** Metabolic fluxes (mmol/g cell N/hour) of cellobiose consumption and product formation in carbon-limited cultures of wild-type C. thermocellum and its PTA deletion mutant (LL1042) grown with excess NH_4_^+^ compared to nitrogen-limited (0.15 g urea/liter) cultivation of the wild type^*a*^

Component or flux^*b*^	Clostridium thermocellum	Clostridium thermocellum (0.15 g/liter urea)	Clostridium thermocellum *Δhpt Δpta*
Cell nitrogen (mg N/liter)	102.0 ± 1.2	40.4 ± 0.2	71.8 ± 2.1
Cell carbon (mg C/liter)	337.3 ± 2.2	183.3 ± 11.1	259.9 ± 10.5
q_Cellobiose_	4.66	9.32	6.61
q_Acetate_	17.34	30.36	0.76
q_Ethanol_	14.89	16.35	25.62
q_Formate_	5.15	7.97	1.66
q_Lactate_	0.38	3.32	1.39
q_Excreted pyruvate_	0.17	**8.56**	**18.19**
q_Total pyruvate_^*c*^	33.03	63.88	51.08
q_Valine_	0.13	**2.64**	**2.56**
q_Excreted amino acids_	89.04	**579.64**	**474.60**
q_Supernatant protein_	101.42	60.04	51.67
q_Nitrogen uptake_^*d*^	9.75	14.26	12.89
Carbon recovery (%)^*e*^	81.8	70.6	79.1

a Amino acid and protein fluxes are expressed as mg/g cell N/hour. Data of the PTA strain were calculated from results of Holwerda et al. (3). Values highlighted in bold represent large increases compared to the values for *C. thermocellum* shown in the leftmost column.

b Cell nitrogen and carbon concentrations are provided as means ± SD.

c q_Total pyruvate_ represents the sum of the fluxes of acetate, ethanol, lactate, excreted pyruvate, and twice the flux of valine in millimoles.

d Nitrogen uptake is calculated by adding N in cells, excreted amino acids and supernatant protein.

e The carbon recovery includes CO_2_ calculated from the amounts of acetate plus ethanol minus formate.

A remarkable phenomenon occurred with respect to the fluxes of amino acids; the flux of excreted amino acids rose 6-fold with increasing C/N ratio in the medium. This increase was mainly due to an increased valine production rate, which rose 20-fold and was accompanied by a 50-fold increase in the flux of excreted pyruvate (Fig. 3C; Table 4). Up to 50% of the consumed nitrogen was excreted as amino acids (Fig. 3C; Tables S3 and S4). Clearly, a dramatic derailment of nitrogen metabolism occurred during nitrogen-limited growth. This phenomenon is also apparent when the gene encoding phosphotransacetylase (PTA) is deleted (3). We calculated that, in the PTA mutant, the specific rate of valine excretion also increased 20-fold, and the specific rate of pyruvate excretion was even 100-fold higher than in wild-type C. thermocellum (Table 4).

## DISCUSSION

### Chemostat cultivation for the study of metabolic fluxes.

The advantage of steady-state analysis of chemostat cultures is obvious; fluxes can be varied at will via the dilution rate, and, contrary to batch cultures, in steady-state cultures, all metabolic fluxes are constant in time. A prerequisite for chemostat cultivation is to define the nature of growth limitation, as this is decisive for the physiology of the microorganism. The results shown in Fig. 1 prove that cultures were cellobiose limited since the fluxes were independent of the inflowing cellobiose concentration and the residual concentration was below the detection limit. Establishing nondetectable concentrations of the intended growth-limiting substrate is not sufficient, as dual limitation for two nutrients may occur (26, 27). This was also observed in our experiments with nitrogen-limited cultures in which dual limitation for cellobiose and urea was observed at a C/N ratio of 12.6 (Fig. 3A; Table 1). Explicit proof of the nature of the growth-limiting substrate is important but not always included in papers involving chemostat cultivation.

In studies on the bioenergetics of an organism, a closed carbon balance is required. In this study, the carbon recoveries were too low for a reliable calculation of the ATP requirements for biomass synthesis (Table 1). The nature of the missing carbon is unknown. Furthermore, considerable amounts of protein and amino acids were present in the cultures (Tables 1 and 2; Tables S3, S4, S5, and S6 in the supplemental material), which makes calculations of Y_ATP_ a complicated enterprise.

### Conversion of PEP to pyruvate.

In C. thermocellum the conversion of glucose-6-phosphate to pyruvate is fully reversible (28) due to the presence of PP_i_-PFK and PPDK (Table 1). The malate shunt (Fig. 2) is thought to be operational as a biosynthetic route for the supply of NADPH via malic enzyme (ME), which is activated by NH_4_^+^ (29, 30) and strongly inhibited by PP_i_ (*K_i_* of 0.036 mM) (30). Lamed and Zeikus (29) reported a half-maximum value for activation of ME for NH_4_^+^ of 0.7 to 0.8 mM for the purified enzyme. This fits with the data of Taillefer et al. (30) for the purified ME from which we calculated an absorption rate constant (*K_a_*) of 0.7 mM based on a Lineweaver-Burk plot. PPDK of C. thermocellum is also activated by NH_4_^+^ (Fig. S2). This explains why, in an earlier study (12), we were unable to detect the enzyme. Using cell extracts, we estimated, in this study, a *K_a_* of 3.8 mM for PPDK, a 5-fold lower affinity for NH_4_^+^ than ME. Therefore, the intracellular concentrations of NH_4_^+^ and PP_i_ are decisive for the division of the flux between the malate shunt and PPDK in C. thermocellum (Fig. 2). Whereas PPDK is reversible, the malate shunt is not because the malate dehydrogenase reaction strongly favors reduction of oxaloacetate to malate. ^13^C-dynamic labeling studies have shown that, in batch cultures of C. thermocellum, two-thirds of the flux from PEP to pyruvate proceeds via PPDK (24).

The importance of a malate shunt for other cellulolytic bacteria is presently not known. In T. saccharolyticum a traditional Embden-Meyerhof pathway is present with an ATP-dependent phosphofructokinase and pyruvate kinase (Table 3). Although PPDK is not essential in T. saccharolyticum (19), its activity was quite high under cellobiose limitation (Table 3). Whether the enzyme contributes to pyruvate formation is not known at present.

### *In vivo* energetics of the PPDK reaction.

**FIG 4:**
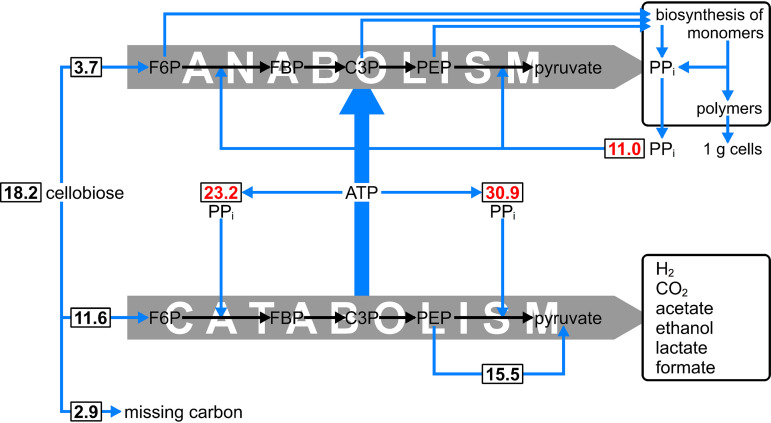
Anabolic and catabolic amounts (mmol) of pyrophosphate and cellobiose in C. thermocellum growing in carbon-limited culture at *D* = 0.1 h^−1^. The anabolic amounts were calculated from an E. coli metabolic network model (32, 33). The total amount of 11 mmol/g cells originates from the synthesis of protein (7.67 mmol), RNA and DNA (1.16 mmol), lipids and lipopolysaccharide (2.11 mmol), and glycogen (0.03 mmol) as reported in reference 12. The catabolic amounts were obtained from the observed biomass yield (0.16 g cells/g cellobiose) from which the sum of unaccounted cellobiose and anabolic cellobiose are subtracted. The amount of PP_i_ required for the conversion of PEP to pyruvate is based on the assumption that one-third of the cellobiose is fluxed via the malate shunt (24).

In the literature, much confusion exists about the impact of a pyrophosphate-dependent glycolysis on the bioenergetics of growth (glucose + 3 PP_i_ + 5 ADP → 2 pyruvate + 5 ATP + P_i_). In many articles, it is assumed that the pyrophosphate in this equation originates from biosynthetic reactions, and no attention is paid to quantitative aspects of the PP_i_ fluxes in the metabolic network. As pointed out previously (12) and shown in Fig. 4, biosynthesis cannot supply enough PP_i_ to satisfy the high demand with the catabolic conversion of glucose to pyruvate in C. thermocellum. Therefore, a mechanism must exist that generates PP_i_ from ATP. For elucidating the impact of a pyrophosphate-dependent glycolysis on cellular energetics, it is therefore important to establish the nature of this PP_i_-generating mechanism. One option would be the reverse reaction of the membrane-bound pyrophosphatase (13–15). However, deletion of the pyrophosphatase gene had no effect on the production of biomass and fermentation products (Table S2). If the nonessential pyrophosphatase was important for the ATP-PP_i_ stoichiometry in the wild type, it should have the same stoichiometry as the alternative PP_i_-generating mechanism that must be present in the pyrophosphatase deletion mutant. The most likely stoichiometry of the alternative PP_i_-generating mechanism is ATP + Pi →ADP + PP_i_ (31).

The effect of a PPDK deletion confirms this conclusion. In wild-type C. thermocellum grown on cellobiose in batch culture, two-thirds of the pyruvate is formed via the PPDK reaction. Deletion of the PPDK gene did not, however, affect fermentation characteristics in batch cultures (24), indicating that *in vivo*, the PPDK reaction does not yield 2 ATP, as pointed out in the introduction. Instead, the absence of an effect of PPDK deletion on biomass yield indicates that the PPDK reaction is energetically equivalent to the malate shunt that produces 1 GTP in the conversion of PEP to pyruvate (Fig. 2). Therefore, *in vivo*, the following overall equation applies for the PPDK reaction:

$$\text{PEP }+\text{ AMP }+{\text{ PP}}_{\text{i}}\to \text{pyruvate }+{\text{ P}}_{\text{i}}\;(\text{PPDK})$$

$$\text{ATP }+{\text{ P}}_{\text{i}}\to \text{ADP }+{\text{ PP}}_{\text{i}}\;(\text{unknown}{\text{ PP}}_{\text{i}}-\text{generating mechanism})$$

$$\frac{2\;\text{ADP}\to \text{AMP}+\text{ATP}(\text{adenylate kinase})}{\text{PEP}+\text{ADP}\to \text{pyruvate}+\text{ATP}}+$$

### Pyrophosphate fluxes in C. thermocellum.

A schematic presentation of the chemostat data of C. thermocellum as presented in Fig. 1 and Table 1 is shown in Fig. 4. In defined media, glycolysis has a dual function: an anabolic one for the synthesis of biomass and a catabolic one for the synthesis of ATP required for anabolism. The amount of cellobiose required in anabolism was calculated from an E. coli metabolic network model (32, 33). It was calculated that 3.7 mmol cellobiose is required for the formation of 1 g C. thermocellum biomass with the same C/N ratio as E. coli and with a carbon content of 45%. The biomass yield was calculated as 1 g cells/18.2 mmol cellobiose. The anabolic and catabolic fluxes of steady-state growth of C. thermocellum are schematically presented in Fig. 4. It has been reported that in the formation of 1 g E. coli biomass 10 to 11 mmol PP_i_ is generated (12, 34). It is evident from Fig. 4 that 11 mmol PP_i_ is not sufficient to sustain the catabolic cellobiose flux in which 23.2 + 30.9 = 54.1 mmol PP_i_ is required when two-thirds of the pyruvate is formed via the PPDK reaction, as estimated for batch cultures (24). It can thus be concluded that an additional mechanism must be present that generates PP_i_ from ATP during cellobiose-limited growth in chemostat cultures.

Most of the pyrophosphate that is formed in biosynthesis results from the first reaction in the formation of peptide bonds between amino acids (aa), the charging of tRNA (12). In organisms like E. coli, the overall process requires the following 4 ATP equivalents:

$$\text{aa1 }+\text{ aa2 }+\text{ 2 GTP }+\text{ ATP}\to \text{aa1}-\text{aa2 }+\text{ 2 GDP }+2{\text{ P}}_{\text{i}}+\text{ AMP }+{\text{ PP}}_{\text{i}}$$

$${\text{PP}}_{\text{i}}+{\text{ H}}_{2}\text{O}\to 2{\text{ P}}_{\text{i}}\;(\text{soluble pyrophosphatase})$$

$$\frac{\text{AMP}+\text{ATP}\to 2\;\text{ADP}(\text{adenylate kinase})}{\text{aa1}+\text{aa2}+2\;\text{GTP}+2\;\text{ATP}\to \text{aa1}-{\text{aa2}+2\;\text{GDP}+2\;\text{ADP}+4\;\text{P}}_{\text{i}}}+$$

**FIG 5:**
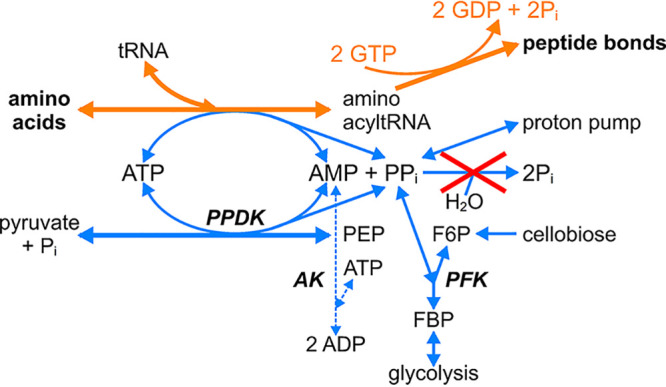
Fate of pyrophosphate in the formation of peptide bonds in the absence of irreversible PP_i_ hydrolysis. AK, adenylate kinase; PPDK, pyruvate-phosphate dikinase; PFK, phosphofructokinase. As a result of the coupling of PP_i_ consumption to reversible reactions, the reaction of aminoacyl-tRNA synthetase may become more reversible. The AMP and PP_i_ produced in the charging of tRNA can be used by PPDK provided that sufficient PEP is converted to pyruvate in the anabolic network.

For the charging of tRNA with an amino acid, 2 ATP are required due to irreversible hydrolysis of the pyrophosphate. According to biochemistry textbooks, this is required to pull amino acids into protein formation, as the charging of tRNA charging is a reversible reaction. C. thermocellum does not possess a soluble pyrophosphatase (Fig. S1). Instead, a membrane-bound proton-pumping pyrophosphatase is present that contributes to the proton-motive force or can be used in the PP_i_-PFK and PPDK reactions. The coupling of PP_i_ production from tRNA-charging with PP_i_ consumption in reversible reactions, such as PP_i_-PFK, PPDK, and the membrane-bound proton-pumping pyrophosphatase, instead of its hydrolysis by a soluble pyrophosphatase, has important metabolic consequences for the anabolic network. Because PP_i_ is not irreversibly hydrolyzed but conserved in reversible reactions, tRNA charging, and thus protein synthesis, becomes weakly coupled to amino acid formation (Fig. 5). As a result, C. thermocellum and other organisms that lack a soluble pyrophosphatase (Fig. S1) may maintain higher intracellular amino acid pools. This, in turn, may contribute to their tendency to excrete amino acids (Table 2) as discussed in the paragraph below.

In the anabolic network of C. thermocellum (Fig. 4), formation of serine and glycine from phosphoglycerate requires 0.5 PP_i_, whereas amino acids and other compounds that are derived from pyruvate require at least 1.5 PP_i_ when produced via the PPDK reaction. For example, formation of valine from 2 pyruvate then requires 3 PP_i_. PP_i_ is even required for the formation of pentose-phosphates from fructose-6P and triose-phosphate due to an alternative pentose phosphate cycle resulting from the absence of transaldolase (35, 36).

$$\text{2 F6P }+2{\text{ PP}}_{\text{i}}\to \text{2 FBP }+2{\text{ P}}_{\text{i}}\;({\text{PP}}_{\text{i}}-\text{PFK})$$

2 FBP→4 C3P (aldolase)

F6P + C3P→C5P + C4P (transketolase)

C4P + C3P→SBP (aldolase)

$$\text{SBP }+{\text{ P}}_{\text{i}}\to \text{C7P }+{\text{ PP}}_{\text{i}}\;({\text{PP}}_{\text{i}}-\text{PFK})$$

$$\frac{\text{C7P}+\text{C3P}\to 2\;\text{C5P}(\text{transketolase})}{{3\;\text{C6P}+\text{PP}}_{\text{i}}\to 3\;\text{C5P}+\text{C3P}}+$$

where F6P is fructose 6-phosphate, FBP is fructose 1,6-bisphosphate, C3P is triose phosphate, C4P is erythrose 4-phosphate, C5P is pentose 5-phosphate, C6P is hexose phosphate, C7P is sedoheptulose 7-phosphate, and SBP is sedoheptulose 1,7-bisphosphate.

Thus, compared to E. coli, the anabolic network of C. thermocellum will produce less PP_i_. The net amount of PP_i_ required or produced in the anabolic network of C. thermocellum is not known because, in existing models, the bulk of the PP_i_ produced in polymerization of monomers to cellular polymers is not quantified but is hidden in so-called growth-associated maintenance (GAM) (37, 38), which assumes hydrolysis of PP_i_ by a soluble pyrophosphatase.

Generation of a substantial amount of PP_i_ via reversal of the pyrophosphatase with a PP_i_-to-ATP ratio higher than unity is unlikely, as the deletion mutant had the same yield of biomass and fermentation products as the wild type (Table S2). When, for simplicity, it is assumed that the 11-mmol PP_i_ generated in anabolism (Fig. 4) is exclusively reconsumed in anabolic reactions, the theoretical amount of ATP required for biosynthesis of cells from glucose (39) will decrease from 34.7 to 23.7 mmol ATP/g cells (i.e., the theoretical Y_ATPMAX_ increases from 28.8 to 42.2 g cells/mol ATP).

An alternative source for the large amount of PP_i_ required in catabolic glycolysis of C. thermocellum (Fig. 4) might be glycogen cycling (12). A key enzyme in the cycle is ADP-glucose pyrophosphorylase (EC 2.7.7.27), which is annotated in cellulolytic *Clostridia* but not in saccharolytic species such as T. saccharolyticum. The overall reaction of the cycle is ATP + P_i_ → ADP + PP_i_. Further investigation is needed to definitively identify the source(s) of nonbiosynthesis-associated PP_i_ in C. thermocellum.

### Excretion of amino acids.

This phenomenon has been observed in both saccharolytic and cellulolytic *Clostridia* (25, 40–42). In culture supernatants of both C. thermocellum and T. saccharolyticum grown on 5 g/liter cellobiose, approximately 100 mg amino acids were present (Table 1). Although lysis may have contributed to this phenomenon, it cannot be the major cause. This follows from the following calculation. With a C/N ratio of 3.34 (Table 1 and Fig. 1), the protein content of cells should be at least 60% (32, 33). Therefore, when originating from lysis the 100 mg/liter (0.9 mM) amino acids present in culture supernatants of both organisms, this amount would originate from 100/0.6 = 167 mg cells (334 μl). Thus, when lysis would be responsible, the intracellular concentration of amino acids should have been 0.9 mmol/0.334 ml, equal to a concentration as high as 2.4 M. It therefore follows that most of the amino acids are derived from excretion by intact cells.

**FIG 6:**
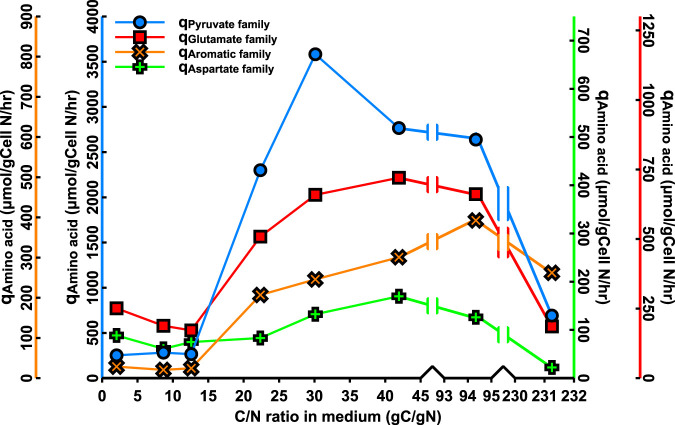
Specific rates of excretion of amino acids of the following four families: the pyruvate family (Ala, Val, Ile, and Leu), the glutamate family (Glu, Gln, Pro, and Arg), aromatic amino acids (Tyr, Trp, and Phe), and the aspartate family (Asp, Asn, Met, Thr, and Lys) as a function of the C/N ratio in the medium feed during growth on cellobiose with urea as nitrogen source.

The increased amino acid excretion under nitrogen limitation is quite unexpected. The large 20-fold increase in the specific rate of valine excretion under nitrogen limitation was associated with a 50-fold increase in the rate of pyruvate excretion (Fig. 3C; Table 4) compared to sugar limitation. This phenomenon is reminiscent of the behavior of PTA deletion mutants of C. thermocellum (3) that exhibited 20-fold and 100-fold increased excretion of valine and pyruvate, respectively (Table 4). In both cases, the common factor is the enhanced cellobiose uptake that apparently leads to an increase in pyruvate accumulation resulting from a shift in the equilibrium of the reversible pyruvate-ferredoxin oxidoreductase (PFOR). In the PTA deletion mutant, this probably results primarily from an increased intracellular acetyl-CoA/CoA ratio since, apart from AdhE, also PTA is responsible for CoA regeneration. We speculate that the excretion of pyruvate and valine under nitrogen limitation results from an increased flux through the malate shunt. The malic enzyme of this shunt has a 5-fold better affinity for NH_4_^+^ than PPDK (Fig. S2), an important factor at very low concentrations of intracellular ammonium that are likely to exist under nitrogen limitation. The malate shunt produces pyruvate and is one of the major sources of NADPH via the malic enzyme reaction. This is in line with increased valine excretion, as the synthesis of valine requires 2 pyruvate and 2 NADPH. An increase in the cellular NADPH/NADP ratio probably affects the reoxidation of reduced ferredoxin generated in the PFOR reaction. The increase in the flux of amino acid excretion does not hold for all amino acids. The fluxes of histidine and lysine decreased with increasing C/N ratios of the medium (Table S5). The largest contributor to the increasing amino acid excretion under nitrogen limitation is due to amino acids in the pyruvate family (Fig. 6), especially valine (Table S5). The different patterns for the various amino acids are an additional argument against lysis as the cause for the presence of extracellular amino acids.

The excretion of massive amounts of amino acids and protein has also been reported for chemostat cultures of Clostridium cellulolyticum growing on cellobiose (42). Interestingly, extremely high NADPH/NADP ratios were encountered in chemostat-grown C. cellulolyticum; at low dilution rates, NADP was not even detectable, whereas at high dilution rates, an NADPH/NADP ratio of 100 can be calculated from the results reported. It remains to be investigated whether in nitrogen-limited C. thermocellum, high ratios are also present. It is evident, however, that a high NADPH/NADP ratio will be a major driving force for amino acid excretion, as the synthesis of amino acids requires NADPH.

### Protein secretion.

Not only amino acids but also significant amounts of protein (100 mg/liter) were present in culture supernatants of both C. thermocellum and T. saccharolyticum (Table 1). Also, this large amount of extracellular protein does not reflect lysis of cells. In C. thermocellum, the synthesis of the cellulosomes attached to the outside of cells is also induced by cellobiose (43). They can detach from cells, and “the cell-free cellulosome complex can be seen as a long-range cellulosome because it can diffuse away from the cell and degrade polysaccharide substrates remotely from the bacterial cell” (44). Also, T. saccharolyticum contains an impressive secretome (45), and it has been reported that most of its very high-molecular-weight endoxylanase during substrate limitation “was extracellular rather than cell associated” (46). The presence of a large amount of secreted protein in chemostat cultures of C. thermocellum and T. saccharolyticum is an additional complicating factor for a calculation of the *in vivo* ATP expenditure for biomass formation.

### Unity and diversity in the metabolism of microorganisms.

A. J. Kluyver presented a lecture in 1924 under this title (47). He classified bacteria (among others, anaerobic cellulose-degrading bacteria) on the basis of the dissimilatory process in their metabolism, even before the discovery of ATP in 1929. He took into account that assimilation and dissimilation are separate processes in microorganisms because of the existence of autotrophic microorganisms. In their pioneering study, Bauchop and Elsden (48) also separated anabolism from catabolism of glucose by using complex media. They verified with ^14^C-labeled glucose that the sugar in their complex medium was used only as an energy source, and they formulated the Y_ATP_ concept. Since then, it has become clear that the proposed 10.5 g cells/mol ATP for anaerobic growth on glucose is not a constant since widely different values ranging from 9 to 23 g cells/mol ATP have been calculated (49). The cause of the gap between the theoretical Y_ATP_ of 29 g cells/mol ATP (39) and the experimentally determined values remains unclear. In their review, Russell and Cook (50) discussed the role of maintenance energy and futile cycling on the biomass yields of anaerobic bacteria grown on glucose. They also could not explain why biomass formation requires much more ATP than can be calculated from the biomass composition by use of textbook biochemistry. Whatever the nature of the missing ATP and the different biomass yields in anaerobic bacteria on glucose, it is beyond doubt that *in vivo* traditional catabolic glycolysis (the conversion of intracellular glucose to pyruvate) yields 2 ATP/glucose.

In our study, we have addressed the question to what extent catabolic PP_i_-dependent glycolysis yields more than 2 ATP. A direct comparison of the energetics of glycolysis between C. thermocellum, which exhibits a mixed acid fermentation, and T. saccharolyticum, which produced practically only ethanol (Table 1), proved impossible due to unacceptably low carbon recoveries and absence of an appropriate *in silico* metabolic network model that specifies the PP_i_ production in, among other things, protein synthesis. In genome-scale metabolic models (37, 38), protein synthesis is incorporated in GAM, and therefore, the pyrophosphate flux remains obscure. From our “on cellulose” model (pencil and paper, Fig. 4), it is evident that the large amount of PP_i_ required in catabolism is not provided by anabolism at no bioenergetic cost. This holds in general for *Hungateiclostridiaceae*, which show a remarkable unity in their biochemistry of glycolysis by using a pyrophosphate-dependent phosphofructokinase (35). Glycolysis via this enzyme must involve a reversible (i.e., a proton-pumping) pyrophosphatase (Fig. S1). The presence of an irreversible soluble pyrophosphatase would be futile and resembles a situation in which both an ATP-PFK and a soluble ATPase are present.

From a theoretical perspective, PP_i_-dependent glycolysis with PPDK must result in a significant reduction in the ATP expenditure in anabolism. Stouthamer (39) calculated that the ATP requirement for the formation of biomass containing 52.4% protein would be 34.7 mmol ATP/g cells. The average molecular weight of amino acid residues in protein is about 110 and therefore in 0.524 g protein, 0.524/110 = 4.8 mmol amino acids are present. With the PPDK reaction, instead of pyruvate kinase, 1 ATP is saved per peptide bond (Fig. 5). Therefore, theoretically, the ATP savings for protein biosynthesis in 1 g cells will be 4.8/34.7 = 14%. In C. thermocellum, the ATP saving is even higher, as with a C/N ratio in cells of 3.34 (Table 1), their protein content will be approximately 65% (32, 33). This theoretical reduction in ATP expenditure in anabolism seems at variance with the observation that a deletion of PPDK does not affect formation of biomass and the regular fermentation products in both C. thermocellum (12, 24) and T. saccharolyticum (19). However, a comparison of biomass yields of wild-type and mutant strains is only valid with a closed carbon and redox balance that also takes the extensive by-product formation into account, especially with respect to formation of extracellular protein and amino acids (Table 1).

The reversibility of the PP_i_-linked glycolysis in C. thermocellum, as proven by labeling studies (28) combined with the absence of irreversible PP_i_ hydrolysis, results in a reversible charging of tRNA (Fig. 5). This probably contributes to the remarkable excretion of amino acids during nitrogen-limited growth (Fig. 6), which is caused by a bottleneck in pyruvate metabolism.

In our study on the turnover of pyrophosphate in C. thermocellum, we distinguished between anabolism and catabolism (Fig. 4). In both processes, PP_i_-PFK and PPDK play a role, but the flux through the catabolic sequence is much higher. The separation of sugar metabolism into anabolism and catabolism is not artificial but is also apparent from the diversity of microbial metabolism. For example, during chemolithoheterotrophic growth of Nitrosomonas europaea, biomass is derived from fructose via PP_i_-PFK, whereas the oxidation of ammonium serves as an energy source (51). Similarly, methanotrophs derive energy from the oxidation of formaldehyde, whereas the assimilation of formaldehyde in species with the RumP pathway often proceeds via assimilatory glycolysis with PP_i_-PFK (52). In these two cases, the amount of PP_i_ required is rather small and may possibly be delivered by only anabolic reactions such as protein synthesis. However, for catabolic pyrophosphate-dependent glycolysis, as occurs in C. thermocellum, this amount is too small (Fig. 4). As the proton-pumping pyrophosphatase is a nonessential enzyme (Table S2), another source of PP_i_ is required. Whether this is indeed glycogen cycling, as observed in cellulose-degrading *Fibrobacter* species (53, 54), remains to be demonstrated.

## MATERIALS AND METHODS

### Strains.

Clostridium thermocellum DSM1313 was acquired from the Deutsche Sammlung von Mikroorganismen und Zellkulturen (DSMZ, Braunschweig, Germany).

Although Clostridium thermocellum has been renamed Ruminiclostridium thermocellum and Hungateiclostridium thermocellum, until a permanent name has been chosen (55), we prefer to use the original name. Thermoanaerobacterium saccharolyticum JW/SL-YS485 (56) was a gift of Juergen Wiegel (emeritus professor, department of Microbiology, University of Georgia, GA) and has been maintained in the Lynd laboratory strain collection since 2008 (21). C. thermocellum was stored under anaerobic conditions at −80°C in 5-ml vials in medium for thermophilic *Clostridia* (MTC), as described in Holwerda et al. (3), with 2 g/liter urea as nitrogen source and 5 g/liter of 3-*N*-morpholino-propanesulfonic acid (MOPS) buffer. T. saccharolyticum was stored in 5-ml vials under anaerobic conditions at −80°C in CTFüD medium (57), prepared as described previously (58), and propagated in CTFüD before being used as inoculum.

Strain LL1639 was derived from strain AG929 aka LL1299 (DSM1313 *Δhpt ΔClo1313_0478*) (4) by deletion of the annotated proton-pumping pyrophosphatase (*Clo1313_0823*) using plasmid pLL1228 (GenBank accession number MT415065).

Fermentation data of strain LL1042 (PTA deletion mutant; Table 4) is available from reference 3, except supernatant protein data, which was measured by Bradford assay (see “Analysis of culture supernatants”).

### Media composition.

The medium used for chemostat cultivation comparing C. thermocellum and T. saccharolyticum was modified from low-carbon medium (59) and contained the following components at a final concentration: 1.25 to 5.0 g/liter cellobiose, 2 g/liter KH_2_PO_4_, 3 g/liter K_2_HPO_4_, 0.1 g/liter Na_2_SO_4_, 2.0 g/liter NH_4_Cl, 0.2 g/liter MgCl_2_·6H_2_O, 0.05 g/liter CaCl_2_·2H_2_O, 0.0035 g/liter FeSO_4_·7H_2_O, 0.025 g/liter FeCl_2_·4H_2_O, and 1.0 g/liter l-cysteine·HCl·H_2_O. Vitamins included 20 mg/liter pyridoxamine hydrochloride, 4 mg/liter 4-aminobenzoic acid, 2 mg/liter d-biotin, 2 mg/liter vitamin B_12_, and 4 mg/liter thiamine hydrochloride. Trace metals included 6 mg/liter MnCl_2_·4H_2_O, 2.5 mg/liter ZnCL_2_, 6 mg/liter CoCl_2_·6H_2_O, 6 mg/liter NiCl_2_·6H_2_O, 6 mg/liter CuSO_4_·5H_2_O, 6 mg/liter H_3_BO_3_, and 6 mg/liter Na_2_MoO_4_·H_2_O. The medium was prepared in 2-liter and 5-liter carboys.

The medium used for investigation of the effect of nitrogen limitation was low-carbon medium and was different from the above-described medium as follows. Five grams per liter cellobiose was used as carbon source, and instead of NH_4_Cl, it contained 2.0, 0.5, 0.35, 0.20, 0.15, 0.10, 0.05, or 0.02 g/liter urea. Na_2_SO_4_ and thiamine hydrochloride were not present.

### Analysis of fermentation products and biomass.

The fermentation products acetate, formate, ethanol, isobutanol, and lactate, as well as cellobiose and glucose, were determined by high-performance liquid chromatography (HPLC) via refraction index. Pyruvate was determined by UV using an Aminex HPC-87H column (Bio-Rad, Hercules CA) on a Waters Alliance HPLC system (Waters, Milford MA) with a 5-mM sulfuric acid solution eluent. Cellular biomass was determined by elemental carbon and nitrogen analysis on a Shimadzu TOC-VCPH total organic carbon analyzer with an added total nitrogen unit and an ASI-V autosampler (Shimadzu Scientific Instruments, Columbia, MD) (60).

### Calculation of carbon and nitrogen recoveries.

Measured fermentation products were used to account for carbon and nitrogen as indicated in the nitrogen and carbon recovery tables. Cell carbon and nitrogen were measured per elemental analysis. Carbon dioxide was accounted for with the following formula (on a mole basis):

$${\text{CO}}_{\text{2}}=\text{acetate}+\text{ethanol}+\text{valine}+\text{ 2}\times \text{ isobutanol}-\text{ formate}$$

The carbon (0.532 g C/g protein) and nitrogen (0.161 g N/g protein) content of protein by weight were as determined by Rouwenhorst et al. (61). In Table 1, the carbon balance for C. thermocellum includes isobutanol, whereas the carbon balance for the same C. thermocellum chemostat data set in Table 4 does not.

### Analysis of culture supernatants.

Residual urea and ammonia were determined by enzymatic kit (urea/ammonia assay kit, Megazyme, Bray, Ireland) against known standards of ammonia and urea. Supernatant protein was determined by Bradford assay (Bio-Rad, Hercules CA, USA) against a dilution series of bovine serum albumin (Thermo Scientific, Rockford IL, USA). Amino acids (alanine, arginine, asparagine, aspartic acid, glutamic acid, glutamine, histidine, isoleucine, leucine, lysine, methionine, phenylalanine, proline, serine, threonine, tryptophan, tyrosine, and valine) were determined from broth supernatant by mass spectrometry as described previously (3).

### Chemostat cultivation.

Chemostat fermentations were performed in jacketed 500-ml custom-made glass vessels with a 300-ml working volume by NDS glass (Vineland, NJ) operated and controlled by a Sartorius Biostat Qplus system (Sartorius, Bohemia, NY). Chemostat cultures were maintained at pH 6.2 for T. saccharolyticum and pH 7 for C. thermocellum by addition of 2 N KOH and with a pH probe (Mettler Toledo, Columbus, OH) and stirred at 200 rpm. During chemostat cultivation, the headspace was purged with 5 ml/min of a N_2_:CO_2_ (80:20) custom-made gas mix (Airgas, White River, VT) for the T. saccharolyticum and C. thermocellum comparison and with 100% ultra-high-purity-grade N_2_ gas (Airgas, White River, VT) for the C. thermocellum nitrogen limitation study. The cultivation temperature was maintained at 55°C with a 19.6-liter PolyStat water bath (Cole Parmer, Vernon Hills, IL). The exhaust gas condensers were kept at 4°C with a 6-liter PolyStat cooling water bath.

After inoculation (2% vol/vol), the batch growth phase was followed by monitoring base addition and optical density at 900 nm (OD_900_) (near-infrared *in situ* OD4 optical probes; Dasgip BioTools, Dasgip/Eppendorf, Hauppauge, NY). The medium-in pump signifying the start of the continuous growth phase was activated for T. saccharolyticum during exponential growth and for C. thermocellum past exponential growth. This proved to be a very reliable method for reaching steady state rapidly. A Watson-Marlow 205S pump continuously administered fresh medium through Masterflex 06404-14 Norprene tubing (Cole Parmer, Vernon Hills, IL). The pump was calibrated and set at a dilution rate of 0.1/hour (residence time of 10 hours). The medium carboy was connected aseptically to the bioreactor and continuously purged with ultra-high-purity-grade N_2_ gas (Airgas) controlled by an FMA mass flow controller (Omega, Stamford, CT) at 2.5 ml/min. The level in the bioreactor was maintained by an effluent pump on the Sartorius control tower activated by closing of an electrical circuit via a level sensor. Effluent was collected in 2-liter waste carboys, placed on scales for continuous effluent weight recording (EJ-6100; A&D, Elk Grove, IL). Chemostats were sampled, and cell material was collected after steady state was established after at least 4 residence times. Results were obtained from steady states separately started and run in 2 chemostats, which were each sampled twice. All data reported in this article are the average of these 4 samples.

### Preparation of cell extracts.

Cells used for enzymatic assays were harvested anaerobically from chemostat cultures at steady state in 50-ml aliquots. Syringes containing the aliquots were closed off aseptically and chilled in a water/ice mixture during transport from the bioreactor to an anaerobic glove bag (Coy Laboratory Products Inc., Grass Lake, MI, USA) immediately to avoid exposure to oxygen. All subsequent handling was done under anaerobic conditions and/or in the anaerobic chamber.

Each 50-ml aliquot of cell culture was pelleted by centrifugation (7,800 × *g* for 10 to 15 minutes). Cell pellets were washed twice with a wash buffer (100 mM Tris-HCl [pH 7.0] and 5 mM dithiothreitol [DTT]), collected in 1.5-ml Eppendorf tubes, pooled under an anaerobic atmosphere in 50-ml Falcon tubes, sealed by anaerobic vinyl tape (471 tape; 3M, St. Paul, MN) to maintain anaerobic conditions, and stored at −80°C upon further use.

To prepare cell extracts for enzyme assays, cell pellets were resuspended in 1 ml wash buffer. Resuspended cells were lysed as described previously (62) by adding 10 μl of 1:100 diluted Ready-Lyse lysozyme solution with 15-minute incubation at room temperature and 2 μl of DNase I solution with an extra 15 minutes incubation at room temperature. The concentration of Ready-Lyse lysozyme solution varied from 20 to 40 kU/μl, and the DNase I solution was 25 U/μl. The crude lysate was centrifuged at 12,000 × *g* for 5 minutes, and the supernatant was collected as cell extract. The total amount of protein in the cell extract was determined per Bradford assay (63) using bovine serum albumin as the standard.

### Enzyme assays.

Enzyme activities were assayed using an Agilent 8453 spectrophotometer connected to a water bath to maintain assay temperature at 55°C in a Coy anaerobic chamber. The reaction volumes for all enzyme assays were 1 ml in reduced-volume quartz cuvettes (part number 29MES10; Precision Cells Inc., NY, USA) with 1.0-cm path length. The units for all enzyme activities are expressed as μmol of product · min^−1^ (mg of cell extract protein)^−1^. For each enzyme assay, at least two concentrations of cell extract were used to confirm that the specific activity was proportional to the amount of extract added.

Activities of glycolytic enzymes were determined as described by Zhou et al. (12) except for pyruvate phosphate dikinase (PPDK) because it has since been discovered that this enzyme requires ammonium for activity. The PPDK assay mixture contained 50 mM Tris-HCl (pH 8.0), 5 mM DTT, 5 mM MgCl_2_, 2 mM AMP, 1 mM PP_i_, 20 mM NH_4_Cl, 4 U/ml lactate dehydrogenase, 0.3 mM NADH, 2 mM PEP, and cell extract. The reaction was started by adding PP_i_.

Isocitrate dehydrogenase was assayed by the formation of 2-oxoglutarate. The assay mixture contained 50 mM Tris-HCl (pH 8.0), 100 mM NaCl, 5 mM DTT, 5 mM MgCl_2_, 1 mM EDTA, 1 mM NAD^+^ or NADP^+^, 1 mM isocitrate, and cell extract. The reaction was started by adding isocitrate.

Glutamate dehydrogenase was assayed by the formation of 2-oxoglutarate from glutamate. The assay mixture contained 50 mM Tris-HCl (pH 8.0), 5 mM DTT, 0.3 mM NADH or NADPH, 1 mM EDTA, 2 mM ADP, 50 mM NH_4_Cl, 5 mM α-ketoglutaric acid, and cell extract. The reaction was started by adding α-ketoglutaric acid.

### Bioinformatics.

Nucleotide sequences were retrieved from GenBank (https://www.ncbi.nlm.nih.gov/genbank/). Identification of homologous protein sequences was performed with BLASTp at the National Center of Biotechnology Information (NCBI). BLASTp was done against nonredundant protein sequences (nr) that were categorized under the *Firmicutes* phylum (taxid: 1239). The protein sequence for the C. thermocellum proton-translocating pyrophosphatase gene, Clo1313_0823, was used to query the database for other similar enzymes; cytosolic pyrophosphatases were identified by querying the database with Escherichia coli Ppa protein, and the Bacillus subtilis YybQ protein was used to query whether family II pyrophosphatases were present. Algorithm parameters were default unless as noted: expect threshold of 10; word size of 3. Default scoring parameters were used (BLOSUM62 matrix; gap costs of 11 for existence and 1 for extension), as well as conditional compositional score matrix adjustments for compositional adjustments. Sequence alignments were performed using the default settings for the online tool T-Coffee (http://tcoffee.crg.cat/apps/tcoffee/do:regular). The alignment supplemental figure was generated with the online tool Boxshade (http://www.ch.embnet.org/software/BOX_form.html).

### Data availability.

Data for strain LL1639 were deposited in GenBank under accession number MT415065 and in the Sequence Read Archive under accession number SRP222605.

## Supplementary Material

Supplemental file 1
