# Supplementary material for: Metabolic Fluxes of Nitrogen and Pyrophosphate in Chemostat Cultures of Clostridium thermocellum and Thermoanaerobacterium saccharolyticum
Source: Appl Environ Microbiol. 2020 Nov 10;86(23):e01795-20. doi: 10.1128/AEM.01795-20 (PMC7657619; doi:10.1128/AEM.01795-20)
Supplement: Supplemental file 1 [file AEM.01795-20-s0001.pdf]

Supplemental Material.

Table S1: Cellular biomass and main fermentation products for chemostat cultivation of *Clostridium thermocellum* at increasing cellobiose concentrations at a dilution rate D=0.1 hr^{-1}. N.D.: Not Detected.

	Cellobiose loading			
Component (mg/L)	1.25	2.5	3.75	5.0
Cell carbon	88.2 ± 1.2	192.3 ± 10.4	263.1 ± 5.3	337.3 ± 2.2
Cell nitrogen	26.4 ± 0.8	60.5 ± 3.3	78.9 ± 1.6	102.0 ± 1.2
Acetate	269.3 ± 0.3	551.7 ± 0.5	791.9 ± 0.4	1062.4 ± 16
Ethanol	96.7 ± 1.5	297.1 ± 2.4	502.9 ± 0.5	699.6 ± 22.2
Formate	23.1 ± 0.5	79.5 ± 0.3	151.2 ± 1.0	241.8 ± 3.6
Lactate	2.8 ± 0.4	19.2 ± 0.2	20.3 ± 0.4	35.3 ± 3.3
Pyruvate	3.6 ± 0.2	12.1 ± 0.5	10.3 ± 0.0	15.4 ± 2.3
Glucose	7.9 ± 0.5	13.0 ± 0.5	16.3 ± 0.2	20.2 ± 1.0
Isobutanol	7.1 ± 0.3	17.2 ± 0.9	23.7 ± 0.8	25.3 ± 0.7
Supernatant protein	14.8 ± 1.3	22.8 ± 6.7	56.6 ± 8.1	103.5 ± 5.7
Cellobiose in feed	1265.9 ± 145.4	2423.6 ± 7.8	3702.3 ± 5.4	4757.2 ± 8.0
Residual cellobiose	N.D.	N.D.	N.D.	N.D.

Table S2: Cellular carbon, nitrogen and fermentation products (mg/L) of strain LL1639 in which the membrane-bound pyrophosphatase gene was deleted (Δhpt Δ PPi-ase as compared to strain LL1299 (Δhpt) from which it was derived. For further information about strains, see Materials and Methods section. N.D.: Not Detected.

Component (mg/L)	LL1299<br>*DSM1313 Δhpt*	LL1639<br>*DSM1313 Δhpt Δ PPi-ase*
Cell nitrogen	93.3 ± 2.6	94.0 ± 1.7
Cell carbon	329.3 ± 6.8	331.7 ± 6.9
Acetate	1033.5 ± 24.3	1060.5 ± 6.1
Ethanol	652.1 ± 15.0	634.3 ± 17.7
Lactate	36.1 ± 4.0	32.2 ± 7.0
Formate	246.6 ± 21.9	253.7 ± 7.9
Glucose	19.7 ± 5.6	23.5 ± 7.2
Pyruvate	21.2 ± 6.8	22.4 ± 6.2
Isobutanol	35.8 ± 1.1	30.1 ± 3.9
CO_2	1194.8	1184.2
Cellobiose in feed	4833.4 ± 33.1	4822.5 ± 30.0
Residual cellobiose	N.D.	N.D.
Carbon recovery (%)	75.5	75.6

Table S3: Physiology of *C. thermocellum* in chemostat cultures at D = 0.1 hr^{-1} grown with a fixed cellobiose concentration of 5 g/L and urea concentrations between 2.0 and 0.02 g/L, the corresponding C/N ratio in the medium is presented between brackets. Concentrations of all compounds are presented as mg/L. CO_2 was calculated from the sum of acetate, ethanol, and twice the amount of isobutanol minus the amount of formate. N.D.: Not Detected.

	Urea in feed (g/L) and carbon to nitrogen ration in the medium.							
Component	*2.0* (2.0)	*0.5* (8.7)	*0.35* (12.6)	*0.2* (22.4)	*0.15* (30.1)	*0.10* (41.9)	*0.05* (94.3)	*0.02* (231.3)
Cell carbon	349.8 ± 20.2	342.5 ± 16.5	334 ± 1.3	235.4 ± 23.4	183.3 ± 11.1	134.2 ± 1.9	80.1 ± 1.7	43.7 ± 0.3
Cell nitrogen	102.6 ± 3.2	98.7 ± 4.7	95.8 ± 0.2	59.4 ± 7.2	40.4 ± 0.2	30.2 ± 0.8	15.9 ± 0.2	6.8 ± 0.7
Acetate	1346.4 ± 27.2	1242.1 ± 17.5	1236.3 ± 20.5	1189.1 ± 217.7	736.3 ± 57.3	491.1 ± 58.1	217.3 ± 15.3	100.9 ± 6.5
Ethanol	482.5 ± 25.0	507.9 ± 27.8	525.5 ± 1.5	443.3 ± 35.9	304.3 ± 25.8	204.6 ± 44.0	73.3 ± 4.2	44.7 ± 12.4
Lactate	59.2 ± 15.1	49.4 ± 10.2	65.3 ± 9.5	110.1 ± 48.2	120.9 ± 17.6	105.1 ± 18.5	33.8 ± 13.4	21.9 ± 1.0
Formate	362.7 ± 26.7	312.0 ± 37.8	333.5 ± 6.3	388.8 ± 156.2	148.2 ± 19.5	97.1 ± 74.5	16.4 ± 23.0	13.5 ± 5.1
Glucose	22.9 ± 14.6	30.3 ± 14.6	24.2 ± 1.4	54.9 ± 3.8	85.1 ± 57.1	138.0 ± 95.9	152.5 ± 149.6	56.1 ± 9.4
Pyruvate	13.4 ± 1.7	17.7 ± 3.3	24.5 ± 3.7	275.2 ± 162.4	304.5 ± 73.2	183.6 ± 122.7	94.7 ± 31.3	41.8 ± 6.8
Isobutanol	15.9 ± 1.3	11.8 ± 0.9	10.8 ± 0.8	22.6 ± 9.9	19.0 ± 2.2	9.7 ± 3.5	N.D.	N.D.
Excreted amino acids	87.7 ± 9.9	72 ± 9.2	71.3 ± 4.1	234.1 ± 101.2	234.1 ± 48.2	152.9 ± 29.1	78.4 ± 17.6	24.5 ± 5.2
Supernatant protein	90.8 ± 6.8	96.9 ± 5.6	115.7 ± 26	36.5 ± 2.3	24.3 ± 3.1	33.4 ± 6.4	33.7 ± 6.5	17.2 ± 2.3
CO_2	1119.6	1111.2	1102.0	950.0	711.1	474.0	213.5	103.8
Cellobiose in feed	4658.0 ± 123.6	4846.0 ± 106.2	4841.7 ± 21.2	4908.8 ± 196.8	4950.3 ± 63.4	4839.7 ± 104.2	4842.4 ± 122.9	4830.5 ± 166.8
Residual cellobiose	N.D.	N.D.	N.D.	45.3 ± 24.4	1185.2 ± 242.8	2010.7 ± 324.6	3303.7 ± 105.7	3878.1 ± 52.5
Urea in feed	2057.0 ± 5.4	504.7 ± 7.6	347.1 ± 1.5	198.2 ± 5.5	148.4 ± 1	104.2 ± 3.2	46.3 ± 6.3	18.9 ± 1.8
Residual urea	868.0 ± 296.0	N.D.	N.D.	N.D.	N.D.	N.D.	N.D.	N.D.
Residual ammonia	494.5 ± 201.8	108.8 ± 7.6	N.D.	N.D.	N.D.	N.D.	N.D.	N.D.

Table S4: Nitrogen balance for chemostat cultures of *C. thermocellum* growing at a fixed cellobiose concentration of 5 g/L and urea concentrations between 2.0 and 0.02 g/L, corresponding C/N ratio in medium is presented between brackets. Nitrogen in protein was derived from 0.161 mg N/mg protein. N.D.: Not Detected.

	Urea in feed (g/L) and C/N ratio in medium							
Component in mg N/L	*2.0* (2.0)	*0.5* (8.7)	*0.35* (12.6)	*0.2* (22.4)	*0.15* (30.1)	*0.10* (41.9)	*0.05* (94.3)	*0.02* (231.3)
Urea	404.9 ± 138.1	N.D.	N.D.	N.D.	N.D.	N.D.	N.D.	N.D.
Ammonium	406.7 ± 165.9	89.5 ± 6.2	N.D.	N.D.	N.D.	N.D.	N.D.	N.D.
Biomass nitrogen	102.6 ± 3.2	98.7 ± 4.7	95.8 ± 0.2	59.4 ± 7.2	40.4 ± 0.2	30.2 ± 0.8	15.9 ± 0.2	6.8 ± 0.7
Supernatant protein	14.6 ± 1.1	15.6 ± 0.9	18.6 ± 4.2	5.9 ± 0.4	3.9 ± 0.5	5.4 ± 1.0	5.4 ± 1.0	2.8 ± 0.4
Amino acids	19.2 ± 5.3	15.3 ± 2.2	14.3 ± 1.3	38.3 ± 18.4	36.4 ± 9.5	25.3 ± 10.8	12.6 ± 3.8	3.8 ± 1.8
Urea nitrogen in feed	959.5 ± 2.5	235.4 ± 3.5	161.9 ± 0.7	92.5 ± 2.6	69.2 ± 0.5	48.6 ± 1.5	21.6 ± 2.9	8.8 ± 0.9
Nitrogen recovery (%)*	98.8	93.1	79.5	112.0	116.6	125.2	156.8	151.3

*Values exceeding 100% may have been due to utilization of cysteine as an additional nitrogen source. Cysteine is present in the medium as a reducing agent (see Materials and Methods).

Table S5: Concentrations of amino acids (mg/L) in chemostat cultures of *C. thermocellum* grown at D = 0.1 hr^{-1} grown with a fixed cellobiose concentration of 5 g/L and urea concentrations between 2.0 and 0.02 g/L, the corresponding C/N ratio in the medium is reported between brackets. N.D.: Not Detected.

	Urea in feed (g/L) and C/N ratio in medium							
Amino acid (mg/L)	*2.0* (2.0)	*0.5* (8.7)	*0.35* (12.6)	*0.2* (22.4)	*0.15* (30.1)	*0.10* (41.9)	*0.05* (94.3)	*0.02* (231.3)
Alanine	7.1 ± 4.4	11.4 ± 3.3	5.5 ± 1.5	8.9 ± 3.6	9.3 ± 3.0	7.6 ± 3.8	3.1 ± 2.3	0.8 ± 0.0
Arginine	N.D.	0.1 ± 0.1	N.D.	N.D.	N.D.	N.D.	N.D.	N.D.
Asparagine	1.4 ± 0.5	1.3 ± 1.0	1.7 ± 0.3	0.1 ± 0.1	N.D.	N.D.	N.D.	N.D.
Aspartic acid	1.7 ± 0.3	1.0 ± 0.2	0.9 ± 0.1	0.7 ± 0.5	0.3 ± 0.1	0.2 ± 0.3	0.1 ± 0.0	0.1 ± 0.0
Glutamic acid	26.8 ± 3.8	20.9 ± 1.1	18.4 ± 1.2	37.7 ± 10.4	31.5 ± 5.2	26.4 ± 18.2	12.6 ± 5.6	3.7 ± 1.0
Glutamine	5.8 ± 6.5	1.5 ± 0.6	1.1 ± 0.2	0.7 ± 0.7	N.D.	N.D.	N.D.	N.D.
Histidine	N.D.	N.D.	N.D.	0.2 ± 0.2	N.D.	N.D.	N.D.	N.D.
Isoleucine	6.0 ± 0.4	5.2 ± 1.0	6.1 ± 0.7	24.7 ± 13.4	29.1 ± 10.4	17.1 ± 0.2	11.4 ± 1.7	3.3 ± 0.6
Leucine	6.4 ± 1.1	3.8 ± 0.7	4.1 ± 0.5	6.5 ± 0.3	7.1 ± 0.4	6.6 ± 1.5	3.5 ± 0.3	1.8 ± 0.0
Lysine	4.7 ± 2.8	1.2 ± 1.0	2.6 ± 0.7	0.1 ± 0.0	0.1 ± 0.1	N.D.	N.D.	N.D.
Methionine	0.1 ± 0.1	0.1 ± 0.1	0.1 ± 0.0	0.9 ± 1.2	4.0 ± 2.0	4.9 ± 6.7	1.4 ± 1.5	0.2 ± 0.0
Phenylalanine	1.8 ± 1.3	1.7 ± 0.3	1.8 ± 0.1	16.9 ± 2.6	13.5 ± 3.6	12.4 ± 7.2	9.0 ± 0.1	6.1 ± 0.6
Proline	4.1 ± 0.5	3.9 ± 0.4	3.7 ± 0.5	4.7 ± 0.9	6.0 ± 0.0	4.4 ± 1.7	2.2 ± 0.7	0.5 ± 0.0
Serine	4.2 ± 0.4	4.5 ± 0.6	5.5 ± 0.3	3.6 ± 1.4	2.2 ± 0.1	1.8 ± 0.3	0.7 ± 0.1	0.2 ± 0.2
Threonine	4.1 ± 0.4	4.0 ± 0.6	3.9 ± 0.1	4.3 ± 1.4	2.8 ± 0.3	2.0 ± 1.4	1.2 ± 0.4	0.2 ± 0.1
Tryptophan	0.8 ± 0.2	0.7 ± 0.3	0.8 ± 0.0	0.3 ± 0.1	0.2 ± 0.0	0.2 ± 0.0	0.1 ± 0.0	N.D.
Tyrosine	2.7 ± 5.6	1.2 ± 0.3	1.5 ± 0.1	3.4 ± 0.6	3.0 ± 0.4	2.7 ± 1.0	1.4 ± 0.2	0.9 ± 0.2
Valine	9.9 ± 1.3	9.5 ± 1.7	13.4 ± 0.1	120.4 ± 91.8	125.0 ± 43.0	66.6 ± 6.7	31.7 ± 4.7	7.2 ± 3.5
Total (mg/L)	87.7 ± 9.9	72.0 ± 9.2	71.3 ± 4.1	234.1 ± 101.2	234.1 ± 48.2	152.9 ± 29.1	78.4 ± 17.6	24.5 ± 5.2
Total carbon (mg C/L)	38.3 ± 12.1	32.1 ± 5.8	32.6 ± 2.9	117.4 ± 64.7	118.0 ± 35.0	76.6 ± 23.0	40.0 ± 8.2	13.4 ± 5.2
Total nitrogen (mg N/L)	19.2 ± 5.3	15.3 ± 2.2	14.3 ± 1.3	38.3 ± 18.4	36.4 ± 9.5	25.3 ± 10.8	12.6 ± 3.8	3.8 ± 1.8

Table S6: Carbon balance over steady states with urea feed concentrations between 2.0 and 0.02 g/L and 5 g/L cellobiose for *Clostridium thermocellum* at a dilution rate D= 0.1 hr^{-1}. The corresponding C/N ratio in the medium is reported between brackets. N.D.: Not Detected.

	Urea in feed (g/L and (C/N ration in medium)							
Component (mg C/L)	*2.0* (2.0)	*0.5* (8.7)	*0.35* (12.6)	*0.2* (22.4)	*0.15* (30.1)	*0.10* (41.9)	*0.05* (94.3)	*0.02* (231.3)
Cell carbon	349.8 ± 20.2	342.5 ± 16.5	334.0 ± 1.3	235.4 ± 23.4	183.3 ± 11.1	134.2 ± 1.9	80.1 ± 1.7	43.7 ± 0.3
Acetate	538.6 ± 10.9	496.9 ± 7.0	494.6 ± 8.2	475.7 ± 87.1	294.5 ± 22.9	196.5 ± 23.2	86.9 ± 6.1	40.4 ± 2.6
Ethanol	251.6 ± 13.0	264.9 ± 14.5	274.0 ± 0.8	231.2 ± 18.7	158.7 ± 13.4	106.7 ± 22.9	38.2 ± 2.2	23.3 ± 6.4
Lactate	23.7 ± 6.0	19.8 ± 4.1	26.1 ± 3.8	44.1 ± 19.3	48.4 ± 7	42.0 ± 7.4	13.5 ± 5.4	8.7 ± 0.4
Formate	94.7 ± 7.0	81.4 ± 9.9	87.0 ± 1.6	101.5 ± 40.8	38.7 ± 5.1	25.3 ± 19.4	4.3 ± 6.0	3.5 ± 1.3
Glucose	9.2 ± 5.8	12.1 ± 5.8	9.7 ± 0.5	21.9 ± 24.4	34.1 ± 22.8	55.2 ± 38.4	61.0 ± 59.8	22.5 ± 3.8
Pyruvate	5.5 ± 0.7	7.2 ± 1.4	10.0 ± 1.5	112.6 ± 66.4	124.6 ± 29.9	75.1 ± 50.2	38.7 ± 12.8	17.1 ± 2.8
Isobutanol	10.3 ± 0.9	7.6 ± 0.6	7.0 ± 0.5	14.6 ± 6.4	12.3 ± 1.4	6.3 ± 2.3	N.D.	N.D.
Excreted amino acids	38.3 ± 12.1	32.1 ± 5.8	32.6 ± 2.9	117.4 ± 64.7	118.0 ± 35	76.6 ± 23.0	40.0 ± 8.2	13.4 ± 5.2
Supernatant protein*	48.3 ± 3.6	51.6 ± 3.0	61.6 ± 13.8	19.4 ± 1.2	12.9 ± 1.7	17.8 ± 3.4	17.9 ± 3.4	9.2 ± 1.2
CO_2**	305.6	303.3	300.8	259.3	194.1	129.4	58.3	28.3
Cellobiose in feed	1961.5 ± 52.0	2040.6 ± 44.7	2038.8 ± 8.9	2067.1 ± 82.9	2084.5 ± 26.7	2038.0 ± 43.9	2039.1 ± 51.7	2034.1 ± 70.2
Residual cellobiose	N.D.	N.D.	N.D.	19.1 ± 10.3	499.1 ± 102.2	846.7 ± 136.7	1391.2 ± 44.5	1633.0 ± 22.1
Carbon recovery (%)	85.4	79.4	80.3	79.9	82.4	84.0	89.8	90.6

* 0.532 gC/gProtein

** CO2 is based on the amount of acetate, ethanol and twice the isobutanol minus the amount of formate produced.

```
DSM 1313      1 MIQITT----------------------------------------QTIQIVAILISLLS
ATCC 27405    1 MIQITT----------------------------------------QTIQIVAILISLLS
DSM 5807      1 MDI-------------------------------------------STALILAIVVSLLS
ATCC 35319    1 MYLQHRQLRKNVLRVLLISLLMGFFVPLSAFAGEADLKTPPLSSTQNSILYIGMLICLLG
DSM 19732     1 ------------------------------------------------------------
DSM 8532      1 MNQ-------------------------------------------TTFELFSVVIAVAA
JCM 21531     1 ------------------------------------------------------------
DSM 8691      1 MNS-------------------------------------------YLLIIYGVVI-IAA

DSM 1313     21 LGVAAWLYSWVKSQPSSNARIAEIGGYIRQGANTFLRREYLILARFTAIAAVLILIFLPH
ATCC 27405   21 LGVAAWLYSWVKSQPSSNARIAEIGGYIRQGANTFLRREYLILARFTAIAAVLILIFLPH
DSM 5807     18 FGVAAYFYIWVKKQPSSNKTVHMVGELIQKGASTFLRREYALLARFAGVIAILILLFLPQ
ATCC 35319   61 MLFGVFQFLKIKK-IAAHKSMLDVADTIYQTCRTYLVQQGKFLGLLLVFIGVCIAFYFGF
DSM 19732     1 ------------------------------------------------------------
DSM 8532     18 FAFAAWLYQWVKKQPSSNKKIAEVGGLIRSGAATFLKKEYTVLARFACVVAILILLFLPH
JCM 21531     1 ------------------------------------------------------------
DSM 8691     17 LAIIGFI-RFIFSQDKGDEKMQQISDSIKEGAMAFLNRQYKTITVLALIVAVIIIIAN--

DSM 1313     81 --PIWSGNITQNIW--------MAVSYIFGTVLSALAGKIGIQIATIANIKSAEAAQKGV
ATCC 27405   81 --PIWSGNITQNIW--------MAVSYIFGTVLSALAGKIGIQIATIANIKSAEAAQKGV
DSM 5807     78 --PIWSGDFKDNIA--------MAVSYIFGTALSALAGKIGITIATIANMKAAEAATKGI
ATCC 35319  120 LQDKGAGGVFLILF--------FTVMGILG---SYSVAWYGIRMNTMANSRMAFSSLER-
DSM 19732     1 -------------------------------------------------IKAAEAATKGV
DSM 8532     78 --PIWQGNAAENIT--------MAVAYLAGTALSALAGKIGIEVATIANVKSAEAAKKGI
JCM 21531     1 ------------------------------------------------------------
DSM 8691     74 ----YYGHQSEGVGSAISYAWHVGLAFISGALCSAISGYIGMYMAVNSNVRAASGARKGL

DSM 1313    131 KP--AFMTGFRGGAVMGMAVVGTSLLGVTLVLLIVGD---A-----------SAVLGFSF
ATCC 27405  131 KP--AFMTGFRGGAVMGMAVVGTSLLGVTLVLLIVGD---A-----------SAVLGFSF
DSM 5807    128 KP--SFLAGFRGGAVMGMAVVGSSLLGVSLVYAITRD---A-----------SAMLGFSF
ATCC 35319  168 KPLKLLSIPLDSGMSIGVILICVELLMMLIILRFVPHELAG-----------AAFIGFAI
DSM 19732    12 KP--SFMTGFRGGAVMGMAVVGTSLLGVTLVIWLVGD---A-----------SAVLGFSF
DSM 8532    128 KP--AFLTGFRGGAVMGMAVVGASLLGVSLVMLITGN---A-----------SAVLGFSF
JCM 21531     1 ---------------MGMAVVGTSLLGVTLVLLLTKD---A-----------SSVLGFSF
DSM 8691    130 NK--ALQIALRGGAVTGLAVTALSLFGVATLFFAYGG---ASGQQELVKNAPSLIVGFGF

DSM 1313    175 GASSLALFAKAGGGIFTKTADISADLVGKVELGLEEDDPRNPAVIADNVGDNVGDVAGMG
ATCC 27405  175 GASSLALFAKAGGGIFTKTADISADLVGKVELGLEEDDPRNPAVIADNVGDNVGDVAGMG
DSM 5807    172 GASSLALFAKAGGGIYTKTADVSADLVGKVELGIPEDDPRNPAVIADNVGDNVGDVAGMG
ATCC 35319  217 GESLGASALRIAGGIFTKIADIGSDLM-KIVFKIKEDDPRNPGVIADCTGDNAGDSIGPT
DSM 19732    56 GASSLALFAKAGGGIFTKTADISADLVGKVELGIEEDDPRNPAVIADNVGDNVGDVAGMG
DSM 8532    172 GASSLALFAKAGGGIFTKTADISADLVGKVELGIPEDDPRNPAVIADNVGDNVGDVAGMG
JCM 21531    32 GASSLALFAKAGGGIFTKTADISADLVGKVELGLEEDDPRNPAVIADNVGDNVGDVAGMG
DSM 8691    185 GASFVALFAQLGGGIYTKAADVGADLVGKVEAGIPEDDPRNPAVIADLVGDNVGDCAGRG

DSM 1313    235 ADLFDSNVASMAAALV--MATALGGPD-SKNVAMVFAYAAIGLLSSVIGVATARI-----
ATCC 27405  235 ADLFDSNVASMAAALV--MATALGGPD-SKNVAMVFAYAAIGLLSSVIGVATARI-----
DSM 5807    232 ADLFDSNVASMVAALV--MAITLDKGA-GNFTMMVFVYAALGLLASSIGVITARM-----
ATCC 35319  276 ADGFETYGVTGVALITFIVLAVTGKLQ-SDMIIWIFTMRILMIVTSIASFYINKLFSKIR
DSM 19732   116 ADLFDSNVASMSAALV--MATSLGGIA-GKNVAMVFIYAAIGLLSSIIGVATARI-----
DSM 8532    232 ADLFDSHVASLAAALV--MAASLGG----SNVNMVLCYAALGLLASIIGVAMARM-----
JCM 21531    92 ADLFDSNVASMAAALV--MATALGGLQ-SKNVAMVFAYAAIGLLSSIIGVATARI-----
DSM 8691    245 ADLFESTAAENIGAMI--LGVGLYPVFGLKGILFPLVARAIGIIASIIGIVFVNTK----

DSM 1313    287 ---GKNGDPGKALNSSTYVTTAIFAVLT------ALSTYIFNFE-----------WRIWG
ATCC 27405  287 ---GKNGDPGKALNSSTYVTTAIFAVLT------ALSTYIFNFE-----------WRIWG
DSM 5807    284 ---GKSGDPSNALNMSTYVTTALYAGAT------ALVTWIFKFE-----------WRIWG
ATCC 35319  335 FADSINIDFEKPLSSLIWITSFLSIIAT------FFASFLMIGPGTVTNESSGGLWLVLS
DSM 19732   168 ---GEKGDPSKALNTSTYVTTGIYAVLT------ALSTYIFGLE-----------WRIWG
DSM 8532    281 ---GKNGDPSGALNNSTYTTTFVYIILT------AIATAVFNFV-----------WRIWF
JCM 21531   144 ---GKDGDPGKALNSSTYVTTAVFAVLT------AVSTYIFGFE-----------WRIWG
DSM 8691    299 ---DESKDPMIALNKGYFVTTIINIIVLFFAVKVMLSGHLSNGDS-------VNYLLLYG
```

```
DSM 1313     327 AT-AIGLIVGTIIGLTSDYFTNDAKPPVRAVANASK-SGPAFTILSGFSYGLLSALPSLI
ATCC 27405   327 AT-AIGLIVGTIIGLTSDYFTNDAKPPVRAVANASK-SGPAFTILSGFSYGLLSALPSLI
DSM 5807     324 AM-IAGLIVGTIIGIASDYFTNDNKKPVHYVAKASE-SGPAFTILSGVSYGFLSVLPALA
ATCC 35319   389 VIISCGTLGAALIPEITKIFTSPKSVHVKETVTASREGGPSLNILSGIVAGNFSAFWKGM
DSM 19732    208 AT-AIGLIVGTIIGITSDYFTNDAKPIVQAVANASK-SGPAFTILSGFSYGLISALPALI
DSM 8532     321 AC-IVGLIVGVIIGIATDYFTNDTKNPVRFVAKASK-SGPAFTILSGMSYGMLSIFPAMV
JCM 21531    184 AT-AVGLIVGTIIGITSDYFTNDARPPVRAVANASK-SGPAFTVLSGFSYGLLSTLPSLI
DSM 8691     349 AA-VAGILISYVFVFLTNYYTSMSTRPVQEIAKAST-TGAATNIITGISVGMESPALPVL

DSM 1313     385 GIAVTALISYYICEPLGS-----GYGMFGISMSALGMLSIVGMIISNDAYGPIVDNARGL
ATCC 27405   385 GIAVTALISYYICEPLGS-----GYGMFGISMSALGMLSIVGMIISNDAYGPIVDNARGL
DSM 5807     382 GIAVSALVAYNLTAQLNPG--NPVYGMFGISMAAVGMLSIVGMIISNDAYGPIVDNARGL
ATCC 35319   449 VFIVL-MFGAYIASRMGLADIMSYPSVFAFGLVAFGFLGMGPVTIAVDSYGPVTDNAQSI
DSM 19732    266 GIAVTSLAAYYICDPLGP-----GYGMFGISMSAVGMLSIVGMIISNDAYGPIVDNARGL
DSM 8532     379 GIAVASLIAYKISEPLGP-----GYAMFGISMSAVGMLSIVGMIISNDAYGPIVDNARGL
JCM 21531    242 GIAVTALISYYICEPLGS-----GYGMFGISMSALGMLSIVGMIISNDAYGPIVDNARGL
DSM 8691     407 FISAAIIIAYRLGELALPN--IATAGFYGTAIATMGMLSTTAYIIAMDTFGPITDNAGGI

DSM 1313     440 AEMGGF--------------------GEKVIAIADELDSAGNTVKAVTKGFSISAAGLTVI
ATCC 27405   440 AEMGGF--------------------GEKVIAIADELDSAGNTVKAVTKGFSISAAGLTVI
DSM 5807     440 VEMGNL--------------------GDKALEITDSLDSAGNTVKAVTKGFAIGAAGLTII
ATCC 35319   508 YELSLIEAIPDIDSEIEKNFGFKPDFEKAKYYLEANDGAGNTFKATAKPVLIGTAVVGAT
DSM 19732    321 AEMGGL--------------------GEKVIAIADELDSAGNTVKAVTKGFSIGAAGLTVI
DSM 8532     434 AEMGEL--------------------GDDVLSITDDLDSAGNTVKAITKGFSIAAAGLTVI
JCM 21531    297 AEMGGF--------------------GEKVIAIADELDSAGNTVKAVTKGFSISAAGLTVI
DSM 8691     465 TEMSEA--------------------PESVRVVTDRLDACGNTTKALTKGYAVGSAALATF

DSM 1313     481 SLLGAFMSEVNTAASELGVQGIANFDIMNPTVFFGLIVGVAIPAVFSAMLMLGVDKNAQR
ATCC 27405   481 SLLGAFMSEVNTAASELGVQGIANFDIMNPTVFFGLIVGVAIPAVFSAMLMLGVDKNAQR
DSM 5807     481 ALLGTFMSEVNVAASERGLAGIENFDILNPTVFFGLLVGAAIPAVFSAMLMLGVDRNAQR
ATCC 35319   568 TMI---FSLILVIKDTLGVKPEEILNLLNPFTILGFLCGGAVIYWFTGASIQAVSTGAYR
DSM 19732    362 ALLGAFMSEVNTAAAELGLETISHFDIMNPAVFFGLIVGIAIPAVFSAMLMLGVDKNAQR
DSM 8532     475 ALLGAFMSEVNSAAAELGKTGIEGFDIINPLVFFGLIIGAAIPAIFSAMLMLGVDRNAQR
JCM 21531    338 SLLGAFMSEVNTAAEELGIQGIMNFDIMNPTVFFGLIVGVAIPAVFSAMLMLGVDKNAQR
DSM 8691     506 LLFSAYLDEVKKILGK-PIDSWFAVDIGKPEVFIGAFIGAMIVYLFSSTAIRAVGKAAQY

DSM 1313     541 MVAEIHR--QWDTIPGLKEGKENAKPDYDKCIDIATTGSLKELIPAGLVAIFSTILVGFI
ATCC 27405   541 MVAEIHR--QWDTIPGLKEGKENAKPDYDKCIDIATTGSLKELIPAGLVAIFSTILVGFI
DSM 5807     541 MVGEIHR--QFNEIPGLKEGKEDAMPEYDKCIDIATTGALKELIPAGLMAIAATIVVGFV
ATCC 35319   625 AVEYIKKNIQLDEDASSKAAVEQSKEVVKICTQYAQKGMVNIFVAVFSFSLAFAFLSAPK
DSM 19732    422 MVEEIHR--QWESIPGLKEGKEGVKPDYDKCIDIATTGSLKELIPAGLTAIIATIVVGFI
DSM 8532     535 MVAEIHR--QFNEIEGLKEGKPGVKPEYDKCIEIATTGAIRELIPAGLMAILATIAVGII
JCM 21531    398 MVEEIHR--QWDSIPGLKEGKDNAKPDYDKCIDIATTGSLKELIPAGLVAIASTILVGFI
DSM 8691     565 VILEVRR--QFKENPGIMEGT--SKPDYAKTVDIVTKGALKEMVIPGLIVVVAPILVGIL

DSM 1313     599 GGASAIGGFL---TGNITSGLLLALLMSNAGGLWDNSKKYVEAGNCGGKGSTAHKAAVVG
ATCC 27405   599 GGASAIGGFL---TGNITSGLLLALLMSNAGGLWDNSKKYVEAGNCGGKGSTAHKAAVVG
DSM 5807     599 GGVNAVGGFL---AGNIISGLLLALFMSNSGGLWDNAKKYVEAGNHGGKGSNAHKSAVVG
ATCC 35319   685 SGNESVALFVSYLVSIAVFGLYQAIFMANAGGCWDNAKKVVEV-DLQQKGTPLHDATVVG
DSM 19732    480 GGVSAIGGFL---TGNIASGLLLALLMSNAGGLWDNSKKYVEAGNCGGKGSPAHKAAVVG
DSM 8532     593 GGVKAIGGFL---GGNIISGLIFALFMSNSGGLWDNAKKYVEAGNEGGKGSDAHKAAVVG
JCM 21531    456 GGVSAIGGFL---TGNITSGLLLALLMSNAGGLWDNSKKYVEAGNCGGKGSTAHKAAVVG
DSM 8691     621 LGKESAAAFL---MVGTISGVIMALFLNNGGGAWDNAKKFIELGNYGGKKSDAHKAGVVG

DSM 1313     656 DTVGDPFKDTAGPSINTQITVVSLVSSLL----------------------ASLFLNVLN
ATCC 27405   656 DTVGDPFKDTAGPSINTQITVVSLVSSLL----------------------ASLFLMYSI
DSM 5807     656 DTVGDPFKDTAGPSINTQVTVVSLVSSIM----------------------ATLFLTIKI
ATCC 35319   744 DTVGDPFKDTSSVALNPIIKFTTLFGLLAMEIAISDTFKDIAPYAGIVLMACALYFVWRS
DSM 19732    537 DTVGDPFKDTAGPSINTQITVVSLVASLL----------------------STLFLLYSL
DSM 8532     650 DTVGDPFKDTAGPSINTQITVVSLVASLM----------------------SALFLTISI
JCM 21531    513 DTVGDPFKDTAGPSINTQITVVSLVSSLL----------------------SSLFLLYSI
DSM 8691     678 DTVGDPFKDTAGPSLHVLIKLISTITLVF----------------------ASLFR----
```

```
DSM 1313     694 FLK----KRNGFALISVA
ATCC 27405   694 F-----------------
DSM 5807     694 FG----------------
ATCC 35319   804 FYKMRVNKEN--------
DSM 19732    575 F-----------------
DSM 8532     688 F-----------------
JCM 21531    551 F-----------------
DSM 8691         ------------------
```

Figure S1. Alignment of annotated proton-translocating membrane-associated pyrophosphatase from several thermophilic bacteria species. Bacteria species (and gene number in parentheses) are as follows, from top to bottom: *C. thermocellum* DSM 1313 (*Clo1313_0823*); *C. thermocellum* ATCC 27405 (*Cthe_1425*); *C. thermosuccinogenes* DSM 5807 (*CDO33_RS16605*); *C. cellulolyticum* ATCC 35319 (*Ccel_2294*); *C. clariflavum* DSM 19732 (*Clocl_0965*); *C. stercorarium* DSM 8532 (*Cst_c10040*); *C. straminosolvens* JCM 21531 (*JCM21531_513*); *T. saccharolyticum* DSM8691 (*Tsac_2336*). Membrane pyrophosphatases were identified by BLASTp, using the DSM 1313 protein sequence for the gene *Clo1313_0823* to query the Firmicutes phylum. A similar BLASTp was performed using the *E. coli* cytosolic pyrophosphatase (Ppa) and *B. subtilis* YybQ to determine whether the abovementioned *Clostridia* species also contained either of these enzymes, but no significant hits were found. BLASTp settings can be found in the Materials and Methods. However, in the case of *T. saccharolyticum*, we observed that the protein product of the *T. saccharolyticum* gene Tsac_0825 produced a significant alignment to the *B. Subtilis* YybQ protein, suggesting at the possible presence of a Family II PPi-ase in *T. saccharolyticum*, although this is not supported with experimental evidence.

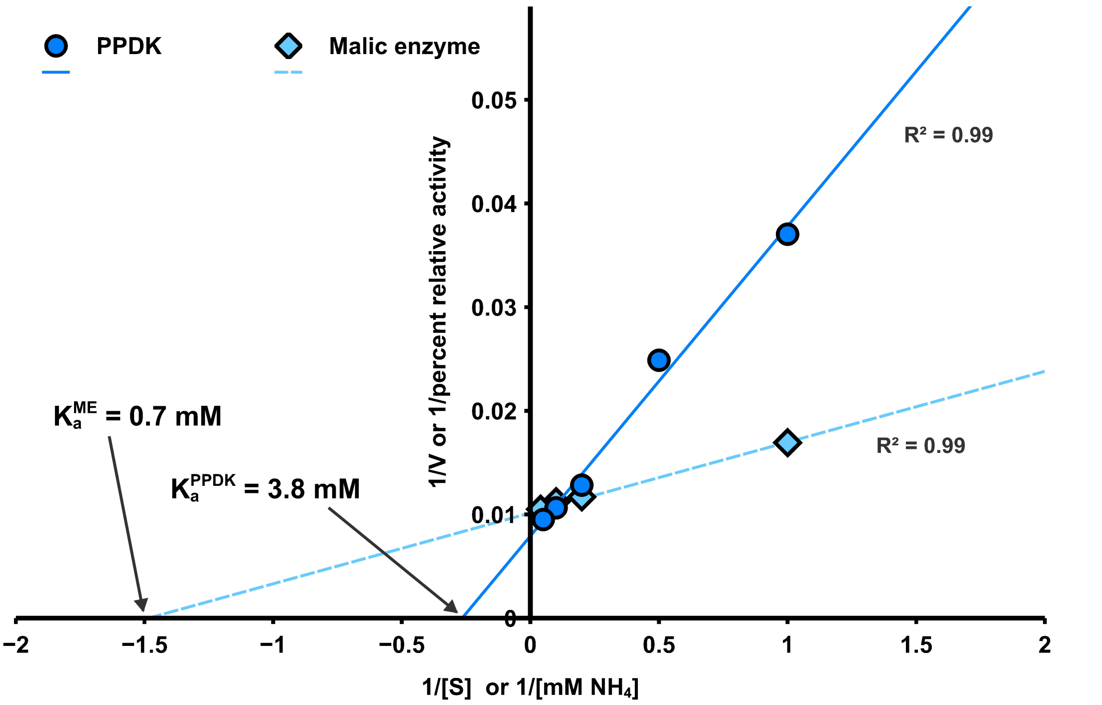

Figure S2: Lineweaver-Burk plots for activation of malic enzyme (ME) and pyruvate phosphate dikinase (PPDK) by NH^+_4. Data for PPDK from Zhou and for ME from Taillefer et al.

Zhou J. 2016. PhD thesis. Comparative analysis of central metabolism in three thermophilic saccharolytic bacteria. Dartmouth College, Hanover, NH.

Taillefer M, Rydzak T, Levin DB, Oresnik IJ, Sparling R. 2015. Reassessment of the transhydrogenase/malate shunt pathway in *Clostridium thermocellum* ATCC 27405 through kinetic characterization of malic enzyme and malate dehydrogenase. Appl Environ Microbiol 81:2423–2432.
